# Impacts of the Deepwater Horizon oil spill evaluated using an end-to-end ecosystem model

**DOI:** 10.1371/journal.pone.0190840

**Published:** 2018-01-25

**Authors:** Cameron H. Ainsworth, Claire B. Paris, Natalie Perlin, Lindsey N. Dornberger, William F. Patterson, Emily Chancellor, Steve Murawski, David Hollander, Kendra Daly, Isabel C. Romero, Felicia Coleman, Holly Perryman

**Affiliations:** 1 University of South Florida College of Marine Science, St. Petersburg, FL, United States of America; 2 University of Miami, Rosenstiel School of Marine and Atmospheric Science, Miami, FL, United States of America; 3 University of Florida, Institute of Food and Agricultural Sciences, Fisheries and Aquatic Sciences Program, Gainesville, FL, United States of America; 4 Florida State University, Dept. of Biological Sciences, Tallahassee, FL, United States of America; Northwest Fisheries Science Center, UNITED STATES

## Abstract

We use a spatially explicit biogeochemical end-to-end ecosystem model, Atlantis, to simulate impacts from the Deepwater Horizon oil spill and subsequent recovery of fish guilds. Dose-response relationships with expected oil concentrations were utilized to estimate the impact on fish growth and mortality rates. We also examine the effects of fisheries closures and impacts on recruitment. We validate predictions of the model by comparing population trends and age structure before and after the oil spill with fisheries independent data. The model suggests that recruitment effects and fishery closures had little influence on biomass dynamics. However, at the assumed level of oil concentrations and toxicity, impacts on fish mortality and growth rates were large and commensurate with observations. Sensitivity analysis suggests the biomass of large reef fish decreased by 25% to 50% in areas most affected by the spill, and biomass of large demersal fish decreased even more, by 40% to 70%. Impacts on reef and demersal forage caused starvation mortality in predators and increased reliance on pelagic forage. Impacts on the food web translated effects of the spill far away from the oiled area. Effects on age structure suggest possible delayed impacts on fishery yields. Recovery of high-turnover populations generally is predicted to occur within 10 years, but some slower-growing populations may take 30+ years to fully recover.

## Introduction

The Deepwater Horizon (DWH) oil spill caused damages across a range of species and habitats in the Gulf of Mexico (GOM). Toxicological effects have been documented in benthic and pelagic fish communities [1,2], estuarine communities [3,4], mammals, birds and turtles [5–7], deep-water corals [8], plankton [9,10], foraminifera [11], and microbial communities [12]. Effects can manifest at the population level as increased mortality or as sub-lethal impairment on the organisms’ ability to forage, reproduce and avoid predators [13]. Second order effects including trophic cascades may take years to reveal themselves and to resolve [13]. These may obfuscate recovery planning, render the ecosystem vulnerable to secondary disturbances, or result in a recovered ecosystem not quite the same as pre-spill conditions. Unfortunately, the fate of the GOM is not easily inferred from previous oil spill experiences due to the unique nature of the DWH oil spill. The scale, the depth, and the subsurface use of dispersants among other factors make this an uncommon case study, but one that may represent the new normal as offshore oil and gas drilling moves into deeper water.

The diversity and complexity of the GOM makes injury quantification difficult, and the National Resource Damage Assessment process has used simplifications to keep the problem tractable, such as focusing on representative species and habitats [14]. Field sampling, laboratory work and modeling has also concentrated on the northern GOM, mainly in the shelf region adjacent to the spill site or within the area of the surface slick and subsurface plume. However, populations far away from the spill area may also be affected, either directly by exposure to oil in the outer margin of their range, or indirectly by the exposure of other species to which they are connected trophodynamically (e.g., prey, competitors, facilitators). For example, post-spill diet changes have been observed in some species [15]. Thus, to understand better the impacts of the oil spill we can take a broader view spatially and taxonomically to factor animal movement and trophic interactions into the calculations of injury and recovery.

**Fig 1:**
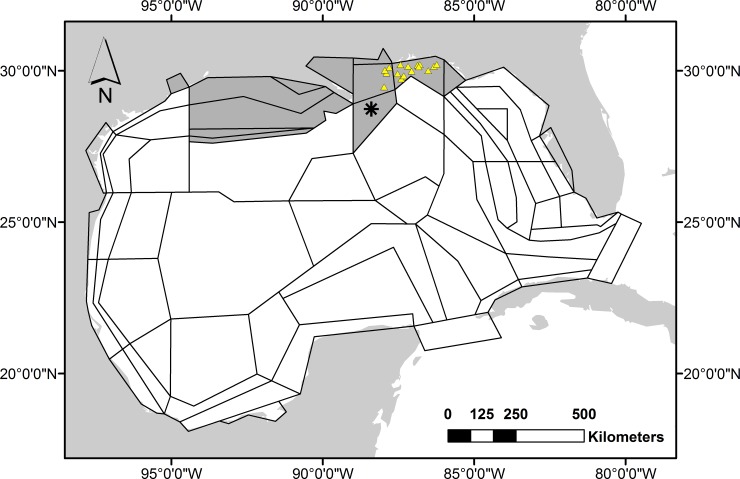
Atlantis polygon geometry. Shaded polygons: heavily impacted areas, asterisk: site of oil spill, triangles: reef survey sites.

In this study, we apply a spatially explicit biogeochemical ecosystem model of the GOM ecosystem (Fig 1) to estimate changes in ecosystem structure with emphasis on eight fish guilds comprising exploited species and their prey. The model, Atlantis, offers a framework on which to synthesize research on the physics, chemistry and biology of the system. Although we have tried to approximate the scale of the oil release and its effects on the biological system, the utility of whole-of-ecosystem simulation modeling is in its ability to account for synergies and antagonisms, and in the study of mechanisms through which unintuitive and non-linear consequences can occur.

We consider changes at the species and community level throughout the GOM. Impacts simulated include lethal and sub-lethal toxicological effects on fish (affecting mortality and growth rates, respectively, as informed by dose-response models), impacts on ichthyoplankton mortality, and impacts of fishery closures. Predictions of the model are validated by comparing population trends and age structure before and after the oil spill with fisheries- independent data (tagging studies and remotely operated vehicle (ROV) surveys). Synthetic indicators such as biodiversity and mean trophic level reveal fundamental changes in ecosystem structure.

## Materials and methods

### Atlantis

Atlantis is a three dimensional spatially explicit deterministic end-to-end ecosystem model. It incorporates ocean physics, chemistry and biology [16–18]. It is coupled to a hydrodynamic model that provides currents, temperature and salinity. These data influence nutrient cycling, primary production and organism physiology and distribution. Flow of nitrogen is tracked through the food web as it passes between functional groups (groups of species aggregated by niche and life history). Each functional group is associated with sub-models describing consumption, production, respiration, reproduction and movement. Interaction rates are modified by a diet matrix, density-dependent feeding relationships, gape limitation, and predator-prey co-occurrence. Vertebrates are age structured and the nitrogen pool is divided into reserve weight (soft tissue and gonadal mass that can be reabsorbed) and structural weight (bone and hard tissues). Human impacts may be represented in several ways including fisheries catch and discards. Seasonal migration into and out of the spatial domain is represented, as well seasonal movement within the domain, vertical movement, density dependent foraging movement and random diffusion. The simulation operates at 12-hour time steps. In this article, we only review model processes most relevant to this application. A more comprehensive review of system equations is available here [16,17]. A summary of applications is available here [18].

The GOM implementation of Atlantis is described in Ainsworth et al. [19]. It incorporates the most recent available data on fish abundance from state and federal survey programs (e.g., from SEAMAP [20]). Biomass was distributed using a statistical methodology [21] and the diet matrix was determined using gut content analysis and statistical analysis [22,23]. Atlantis polygon geometry is based on bioregional features (e.g., physical processes, habitat, and climatology), exploitation patterns and management jurisdictions [19]. The model uses 91 functional groups: 61 are age-structured vertebrates (including fishes, birds, turtles and mammals), 19 are invertebrates, 6 are primary producers, 2 are bacteria and 3 are detritus. Model functional groups represent single species in the case of important fishery targets, or aggregated groups of species with similar life histories, distributions and feeding patterns. Vertebrates and some exploited invertebrates are tracked by numbers and body weight, while non-exploited invertebrates are handled as biomass pools. This allows us to represent processes and rates relevant to fisheries management (e.g., size-based gear selection, fecundity at age) and it minimizes problems arising from data quality in poorly studied species. Polygon geometry reflects circulation, habitat and management divisions (Fig 1). There are up to 6 water column layers per polygon, plus a sediment layer. Initial conditions represent the ecosystem in January 2010. Hydrodynamics are provided by the American Seas model (AMSEAS) based on the National Research Laboratory-developed NCOM model [24].

### Growth and mortality effects

In Atlantis, the instantaneous rate of change in biomass (B, in units mg N) for each polygon, depth layer and age class of functional group *i*, is determined by Eq 1.

$$\frac{dB_{i}}{dt}=[R_{i}+T_{IMM,i}-T_{EM,i}-{M1}_{i}-\sum_{j}P_{i,j}-F_{i}]∙g_{oil}[G_{i,s}+G_{i,r}]$$ (1)

All rates are specific to each polygon and depth layer except for M1 and g_oil_. T_IMM,*i*_ and T_EM,*i*_ are immigration and emigration, F_*i*_ is fisheries catch, and P_*i*,*j*_ is predation by predator *j* on group *i*. G_i,s_ and G_i,r_ are growth in structural and reserve nitrogen (these are functions of assimilation efficiency, growth rate and total consumption). R is entry of new individuals into the age class from recruitment or aging. In this application, M1 summarizes natural mortality from non-predation sources as in Eq 2.

$$M1=m_{l}+m_{q}+m_{oil}$$ (2)

Here, *m*_*l*_ and *m*_*q*_ are linear and quadratic mortality parameters set iteratively during model calibration and *m*_*oil*_ is mortality derived from the dose-response model in Eq 3. *g*_*oil*_ is the modifier on growth rate caused by oil exposure in Eq 4.

$$m_{oil}=m_{pelagic}∙(1-B)+m_{benthic}∙B$$ (3)

$$g_{oil}=g_{pelagic}∙(1-B)+g_{benthic}∙B$$ (4)

The *m*_*oil*_ term represents a weighted average of a pelagic mortality modifier (*m*_*pelagic*_) and a benthic mortality modifier (*m*_*benthic*_). The weighting reflects the proportion B of the affected group’s diet that comes from benthic prey. This serves as a proxy for how intimately a functional group associates with contaminated sediments. Similarly, *g*_*oil*_ is a weighted average of pelagic and benthic growth rate modifiers.

Modifiers are calculated via the dose-response models in Eqs 5 and 6 (note change in symbols: e.g., *m*_*t*_ = *m*_*pelagic*_ at time *t* when the amount of bioavailable oil *φ*_*t*_ is representative of the pelagic environment). The dose-response models were developed in a previous publication [25]. They are based on organism responses to petrogenic PAHs from exposure studies and field sampling of the DWH oil spill and elsewhere. Lesion and tumor frequencies were used as proxies for changes in mortality rate and otolith annuli measurements were used to infer growth rate impacts. Due to the limited available ecotoxicology data, it was not possible to develop mortality and growth responses specific to Atlantis functional groups. The mortality response was based on 106 species from 40 fish families and the growth response was based on nine species from six families [25]. These models were applied to all fish functional groups, assuming a similar physiological vulnerability to oil toxicity, although functional groups varied in their level of exposure according to the oil transport model described below.

The ‘hockey stick’ dose-response models in Eqs 5 and 6 were selected from among linear, exponential and step-function models using an Akaike information criterion. The hockey stick model implies that there is a PAH concentration threshold below which there are no adverse effects and above which there is a log-linear decrease or increase in growth or mortality rates, respectively. The effect size for mortality (*m*_*t*_) and growth (*g*_*t*_), vary with time step *t*.

$$m_{t}=\alpha ∙\log\left[K\varphi_{t}∙\frac{1}{\beta }\right]∙\omega^{-1}$$ (5)

$$g_{t}=\gamma ∙\log\left[K\varphi_{t}∙\frac{1}{\delta }\right]∙\omega^{-1}$$ (6)

Parameters *α* and *γ* are slopes and *β* and *δ* are the PAH concentration thresholds where an effect manifests. These were fit by maximum likelihood estimation [25] (*α* = 0.2885, *β* = 907.4306, *γ* = 0.0531, *δ* = 28.422). Since experiments exposed animals to oil over the course of one or more weeks, a mean exposure time of *ω* = 15 days is assumed so that we may approximate the daily effect. Note that we do not necessarily assume direct mortality from oil, but rather an increase in the likelihood of mortality from any source due to reduced health and behavioral changes [26]. Although certain components of the oil are known to be more toxic than other components, there are too few response data available to discern toxicity based on oil composition. Dose response models for mortality and growth are assumed applicable to all fish functional groups and age classes (although total effect size is modulated by sediment association as in Eqs 3 and 4 and by co-occurrence of oil and life stage distribution). We assumed that no avoidance behavior occurred.

Organisms used in lab and field exposure studies to develop the dose-response models were typically from chronically oil-exposed areas [25]. Since resistance has been documented in fish populations to a range of organic pollutants, either through genetic adaptation or physiological acclimation [27], we acknowledge that fish populations exposed to DHW oil could react more severely to oiling than those examined in exposure studies. Therefore, we conduct sensitivity analysis on the threshold parameter of the hockey stick model, reducing the threshold parameter *β* (by -0%, -20%, -40%, and -60%) for simulations involving the mortality modifier. These simulations are referred to as *β*907, *β*726, *β*544, and *β*363, respectively (the numerical value corresponds to the PAH threshold in ppb). The threshold for growth effects is already low (*δ* = 28.4 ppb) so a sensitivity analysis is of less value on this parameter.

Exposure studies used to build the dose-response relationships consistently reported PAH concentrations in the sediment [25]. Unfortunately, we did not have enough sediment oil data from the field to use directly as the oil driver in the simulations. The few sediment oil data that we had existed for only small and non-contiguous areas of the GOM (I.R. and D.H., unpublished data). However, we had water column data for the entire GOM from oil transport modeling. We therefore inferred what the sediment concentrations must have been based on the water column data. To do this, we multiplied time- and depth-integrated water-column oil concentration values by a sediment-to-water column ratio (K). This ratio was informed by a comparison of oil transport modeling results with field-collected sediment cores sampled by C-IMAGE (I.R. and D.H., unpublished data). There is potentially a concentration factor up to 1000 times (S1 Fig). In reality, sedimentation varies by suspended particulate load and microbial activity over the study area, as well as by variations in oil density in the water column and by the sediment composition. These factors are not taken into account by our simple empirical ratio. However, Atlantis polygons are more than 25,000 km^2^ on average and include areas of both below- and above-average deposition rates, thus small-scale spatial variations can be ignored. Persistence of the sedimentary oil is managed by a depuration model, explained below.

We vary the concentration factor (K) in sensitivity analysis (by -0%, -20%, -40%, and -60%) for simulations involving mortality and growth modifiers. Hereafter, these simulations are referred to as K1000, K800, K600, and K400 respectively. Therefore, our sensitivity analysis consists of 16 oil spill simulations varying K and *β*. Where results from a single oil spill simulation are shown, we indicate the worst-case scenario, [K1000 *β*363]. This offers an upper bound for potential impacts under our assumed conditions and it should make qualitative effects on ecosystem structure more apparent.

Benthic and pelagic modifiers in Eqs 3 and 4 are calculated in the same way, but they assume different amounts of bioavailable oil *φ*_*t*_ at time *t*. This amount is determined by an uptake/depuration model that implicitly represents accumulated body burden (Eq 7).

$$\varphi_{t}=O_{n,t-1}∙\frac{E_{t}}{N}∙\sum_{n}^{N}\left(\mu ∙O_{n,t}\right)∙e^{-\rho }$$ (7)

Hydrocarbons may accumulate through direct uptake from the water by gills or skin, uptake of suspended particles or through ingestion through food. In all cases, there is potential for bio-magnification of toxins. We have combined all processes into a single uptake function, where the rate of uptake is the product of an uptake constant (*μ* = 1 for benthic environments and *μ* = 0.1 for pelagic environments) and pollutant load *O*_*i*,*t*_ as determined from oil transport modeling. In the depuration constant *ρ*, we have summarized into a single term the effects of gill elimination, metabolic transformation, fecal egestion, growth dilution and elimination via egg deposition and sperm ejection [28]. A rate of depuration is assumed that achieves 99% clearance in 20 days (*ρ* = 0.2424). Note that ecotoxicology experiments will improve this estimate (D. Wetzel, Mote Marine Laboratories).

### Oil transport and fate modeling

Oil concentrations were taken from a probabilistic framework for oil droplet-tracking based on the Connectivity Modeling System (CMS) [29], an open-source Lagrangian stochastic model. Examples of oil applications of the CMS (oil-CMS) are provided by Paris et al. [30]. The geographical distribution and depth of the so-called deep plume is similar to observations by Kessler et al. [31]. Surface oil is informed by Le Hénaff et al. [32] and validated against remote-sensing observations. However, the configuration and oil concentration calculations of the model applied here have improved over Paris et al. [30] since we have used published values of the actual oil spilled [33,34]. Degradation dynamics now consider new results from high-pressure experiment data [35,36] and hydrocarbon fractionation is implemented more realistically with all fractions in a single droplet, allowing dissolution (C.B.P. and N.P. unpublished).

The oil-CMS computes the 3-D oil particle trajectories and their evolution. Its fourth order Runge-Kutta advection scheme utilizes 3-D momentum, temperature and salinity data from the high-resolution ocean model with data assimilation. The oil-CMS estimates droplets’ terminal velocity depending on their size and density and on ambient fluid properties (temperature, salinity, density and viscosity). Additional considerations account for oil dissolution, biodegradation rates and surface evaporation [35]. The initial conditions of the model are specified a priori. The most recent simulations of the Deepwater Horizon incident are summarized in an article in preparation (C.B.P. and N.P., unpublished manuscript). Briefly, we used an initial oil droplet size distribution of 1–500 microns [33]. Post-processing analysis of modeled oil droplet location and properties through time translated the model output data into oil concentrations. This used estimates of the actual oil spilled [34,36] and the assumed droplet size distribution at the time of the release [37].

CMS operates at 1/25^th^ degree horizontal resolution over 20 vertical layers. The oil droplets are released bi-hourly for 87 days and tracked for 167 days in the area bounded by 25^o^N and 30.75^o^N latitude, 93^o^W and 84^o^W longitude. Oil concentrations are integrated over the vertical dimension to match Atlantis’ depth layers (partitioned at 10 m, 20 m, 50 m, 200 m and 2000 m) and provided to Atlantis at 24-hour intervals. Dose-response calculations are made at each horizontal grid point and averaged over Atlantis’ polygons, adjusting for the proportion of grid points per polygon that do not contain oil. We use a 100-day ‘spin-up’ time before introducing oil in the Atlantis simulation to allow transient biomass dynamics to settle. Oil forcing lasts for 167 days and depuration continues thereafter according to Eq 7.

### Fisheries closures

We conduct model runs that simulate DWH oil spill emergency fishery closures as spatiotemporally dynamic fishing closures. The schedule is reported by the National Centers for Environmental Information [38]. ArcGIS shapefiles [39] for closures were processed relative to the Atlantis polygon map using an Intersect tool and the proportion of each polygon overlapped a closure was closed to the appropriate fishing fleet(s) in Atlantis. This reduces local fishing mortality proportionately without reallocation of spatial effort. We simulate closures from April 20, 2010 to April 19, 2011, the duration of the emergency closures, updating spatial configuration at daily time steps.

### Recruitment

Additional simulations test potential impact on recruitment. Taxon-specific impacts on fish larvae were calculated in a previous study based on the overlap of larval fish distributions and observed surface oil from the DWH oil spill [40]. Distributions of larval fish were created using 27 years of samples collected by the Southeast Area Monitoring and Assessment Program (SEAMAP). Recorded counts were standardized to create monthly average abundance distributions. Surface oil distributions were provided by a study conducted by NOAA, which binned the surface oil features on a daily basis using the presence/absence of oil and a semi-quantitative estimate of oil thickness (density). See Murawski et al. [1] for further explanation. The estimated proportions of fish larvae potentially exposed to DWH oil were calculated as the abundance of fish larvae located within the oiled area during the months of April through July divided by the total abundance of fish larvae throughout the entire year in the northern GOM study area. It is assumed that any larva exposed to oil was killed [40].

### Analysis

Unless otherwise stated, all results shown are averaged over the shaded polygons in Fig 1. Out of the 64 dynamic polygons in the model, these 12 showed the greatest proportional change in biomass (averaged across fish functional groups) due to the oil spill (i.e., relating to the net effects of processes such as direct toxicological impacts, seasonal or ontogenetic movement of impacted populations, or movement of impacted prey resources). These areas also roughly correspond to areas of injury assessment [14] efforts. For clarity, we have aggregated results from Atlantis fish functional groups into eight ‘guilds’: snappers (Family: Lutjanidae), groupers (Family: Serranidae), Sciaenidae, elasmobranchs, large pelagic fish, small pelagic fish, small demersal and reef fish, and large demersal fish. Atlantis functional groups that comprise these guilds are presented in S1 Table. Species that comprise Atlantis functional groups are presented in Ainsworth et al. [19]. Simulations are 50 years’ duration, from 2010 to 2060.

We compare Atlantis’ predictions on age structure changes against tagging data from Southeast Data, Assessment and Review (SEDAR) reports. We also compare Atlantis’ predictions on species composition and body size changes against ROV fish count data and laser-scaled fish size estimates. Comparisons against ROV data are done at the level of functional groups. There were sufficient ROV abundance data to compare against 16 model functional groups. There were sufficient ROV size data to compare against 9 model functional groups. The ROV data are from 16 natural reefs sites on the northern Gulf shelf located from Mobile Bay to Choctawhatchee Bay (W.P., unpublished data) (Fig 1). The sites range from 17 to 75 m depth. Observations were made between August 2009 and Sept 2015 and so they represent pre- and post-spill conditions. Since the fish numbers from Atlantis represent an average over oiled polygons, they encompass a full range of age classes, habitats and depths. It is not meaningful to compare densities with ROV surveys in absolute terms since model data represents the average over polygons. Instead, we concentrate on trends, scaling median values to match the ROV data. Laser-scaled fish size estimates were converted to body weight based on length-weight relationships in Fishbase [41]. This comparison requires a caveat that only a fraction of species and age classes constituting these functional groups are present in surveys.

## Results

### Fisheries closures and recruitment effects

The model suggests that fisheries closures and loss of larvae due to oil exposure (hereafter called recruitment effects) have little impact on ecosystem biomass (S2 Fig). Closed areas are responsible for a minimal biomass increase; no more than 1/3 of 1% in any species guild relative to a no-oil scenario (see S1 Table for guild compositions). Recruitment effects similarly have a negligible impact. When averaged across Atlantis functional groups, the recruitment impact equated to a 5.8% (σ = 4.5%) loss of the larval population in the year of the oiling. This was sufficient to reduce biomass only about 1/10^th^ of 1% for any guild. Across species guilds, the MPA effects were responsible for no more than 3.6% of the variance of biomass, and the recruitment effects were responsible for no more than 0.2% (both measured monthly from spill up until to one year post-spill). As modeled, these simple treatments result in short-lived effects that do not impact long-term recovery. Therefore, we omit fisheries closures and recruitment effects from subsequent analysis to concentrate on direct and indirect effects of oil toxicity on juvenile and adult life stages.

### Impacts on biomass

**Fig 2:**
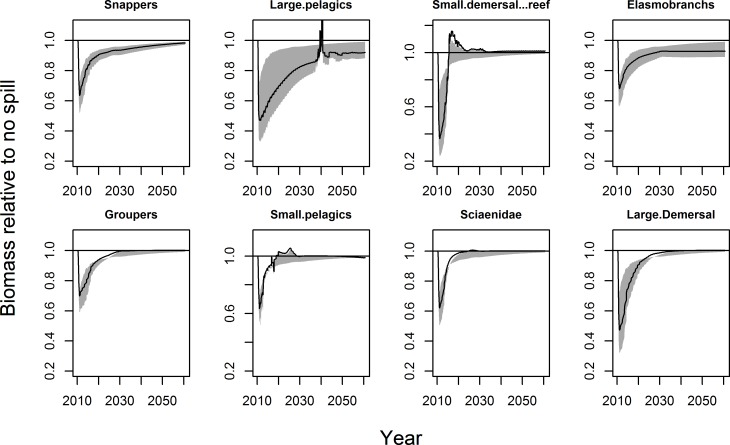
Biomass trajectories for species guilds. Biomasses are summed across all functional groups within these guilds. Shaded area shows range of outcomes observed in sensitivity analysis on concentration factor K and threshold *β*. Black line shows the mean of the 16 sensitivity runs.

Fig 2 shows biomass in the oil scenarios relative to the no-oil scenario for 8 species guilds including the mean and range of variation predicted under different settings of the assumed water column-to-sediment concentration factor K and oil-effects threshold *β*. The largest decreases in biomass relative to a no oil scenario occur within 7–16 months after the spill (median 10 months). Guilds comprised of large reef fish (i.e., snappers, groupers and sciaenids) as well as elasmobranchs and small pelagic fish exhibit a major impact, decreasing by 25–50% biomass relative to the no oil scenario in the most heavily oiled areas. This closely matches observed declines from ROV surveys conducted before and after the spill (see section *Comparison against ROV survey data*). Large impacts also occur in large pelagic fish and large demersal fish. The model suggests decreases in those guilds by 40–70%. A larger effect occurs in small demersal and reef fish. This guild, which constitutes the benthic and reef forage base, decreases by 50–75%. S2 Table provides biomass minima (i.e., minimum biomass reached by guilds throughout the simulation) for all variations of K and *β*. Note that small differences between the guilds are not meaningful in light of the error range of the model, but it can be said that small demersal and reef fish exhibit the largest effect followed by large pelagic fish and large demersal fish.

### Recovery

**Fig 3:**
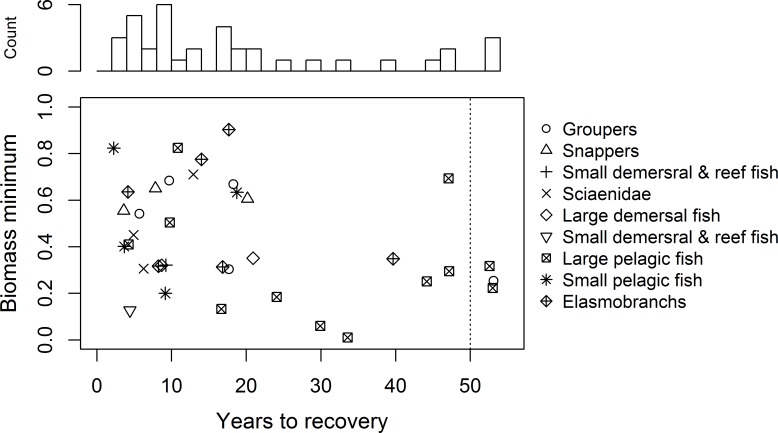
Biomass minima versus years to recovery. Data are relative to no-oil scenario. Criterion for recovery: achieving 99% of biomass of the no-oil scenario. Functional groups > 50 years to recovery did not recover within simulation period. Points show Atlantis functional groups arranged by guild. Represents oil simulation [K1000 *β*363].

There is no consistent relationship between biomass minima and recovery time (Fig 3) but small-bodied fish recover the fastest. For example, the small demersal and reef fish guild and most of the groups constituting the small pelagic fish guild return to pre-spill conditions within 10 years. One group each from the elasmobranchs, snappers, and groupers guilds takes longer than 20 years to recover. Three functional groups did not recover within the 50-year time horizon: two large pelagic fish and a grouper (S1 Table). More than half of the Atlantis functional groups comprising the large pelagic fish guild take longer than 30 years to recover. The large pelagic fish guild was also among the most heavily impacted guilds in terms of biomass lost (Fig 2). However, this finding is not borne out by commercial catch statistics (S3 Fig). This discrepancy is informative and we will return to it later.

### Age structure

**Fig 4:**
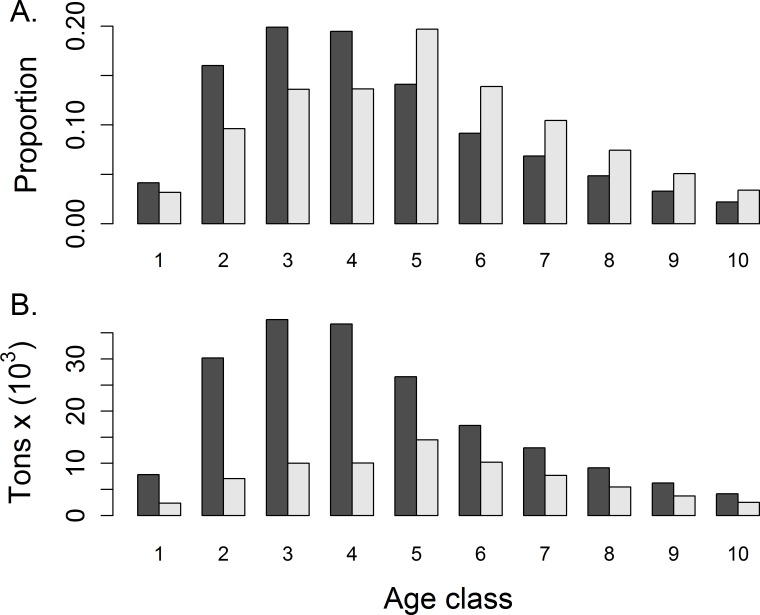
Differences in age composition for the large demersal fish guild. No oil (dark gray); oiled (light gray). Data represent October 2010 for heavily impacted polygons. Represents oil simulation [K1000 *β*363]. A) Relative proportion, B) absolute biomass.

Reef-associated and demersal fish guilds experienced a loss of young individuals with the oiling event and therefore an immediate shift in age structure towards older individuals (S4 Fig). This is typified by the large demersal fish guild (Fig 4). This trend is supported by aging studies for almost every species assessed in the GOM since 2012 (S5 Fig, S3 Table). Only Gulf menhaden age composition fails to follow this trend. However, within a few years after the loss of these young fish, the model predicts a depressed number of reproductive aged individuals relative to the no-oil scenario, and therefore a shift towards a relatively younger age structure that persists for 5 or 10 years for most guilds (S6 Fig).

### Spatial patterns

**Fig 5:**
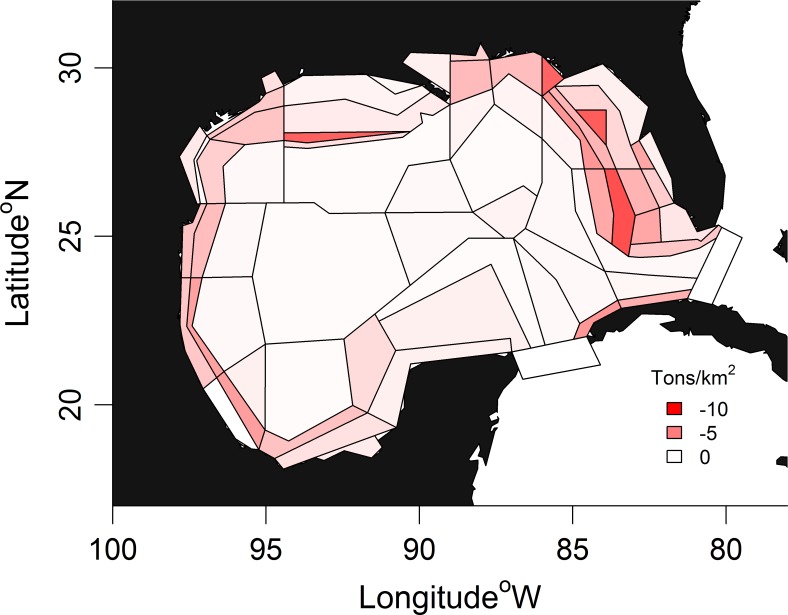
Absolute biomass reduction for no oil versus oil scenario for grouper guild. Biomass minima is shown occurring at 10 months after the oil spill. Oil simulation [K1000 *β*363].

**Fig 6:**
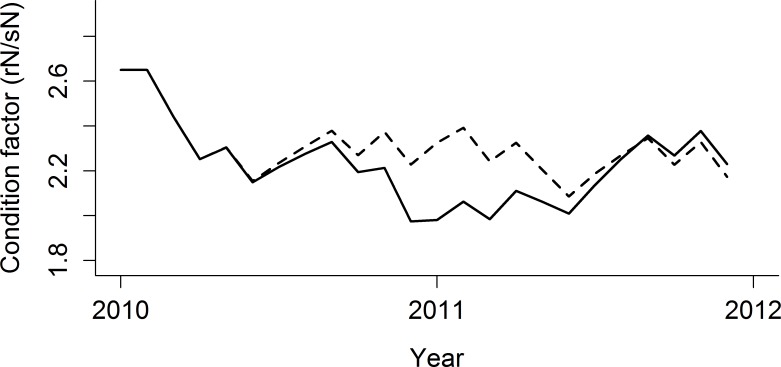
Condition factor of grouper guild. Condition factor is represented as reserve:structural Nitrogen ratio. High rN/sN indicates good body condition. Dotted line: no oil scenario, solid line: oiled [K1000 *β*363].

Ecological impacts from the DWH oil spill manifest at great distances away from the slick and sub-surface plume. The spatial pattern of impact for groupers (Fig 5) is typical for reef and demersal species (S7 Fig). Reduced biomass is predicted on West Florida Shelf populations, particularly in the south, and as far away as the Texas shelf and Campeche Bay. This can be attributed to impacts on the mobile prey base and/or on prey populations interconnected by larval transport. Such indirect trophic interactions have been shown to influence recovery dynamics with previous oil spills [42]. A decrease in condition factor (Fig 6) verifies that groupers are unable to consume enough prey for their needs despite an increase in the per capita rate of consumption (S8 Fig). Condition factor is represented as the ratio of reserve (soft tissue) to structural Nitrogen (hard tissue). The decrease in grouper condition factor is relatively large. During the period when condition factor is lowest in the oiled scenario (black solid line in Fig 6, around 2011), the grouper guild’s condition factor is 2.01, which is equivalent to the 32^nd^ percentile of all functional groups’ pre-spill values. However, without the oil effect, the grouper guild’s condition factor would be 2.32 at the same point in time, equivalent to the 60^th^ percentile of functional group pre-spill values. Reduced condition factor is predicted in all guilds to some degree (S9 Fig).

### Changes in the food web

Since the demersal and reef forage base was impacted more severely than the pelagic forage base, the model predicts a shift towards pelagic prey relative to demersal prey for all guilds (S8 Fig). The guilds are broad enough taxonomically to include some opportunistic feeders. At the same time, the model predicts an overall increase in the per capita consumption rate since so many predators have been eliminated (Fig 2). In some cases, the increase in per capita consumption rate may be partly due to the shift in age structure in predators towards (more voracious) young individuals, which happens subsequently to the initial loss of young individuals due to oiling (S6 Fig). The increased abundance of pelagic forage relative to demersal forage is indicated in the ecosystem’s increased pelagic-to-demersal ratio (S10 Fig). Other ecosystem indicators reveal structural changes in the food web. Major impacts on the forage base results in an increase in the ecosystem’s piscivory to planktivory ratio, while the loss of dominant predator functional groups results in system-wide decreases in mean trophic level and Shannon’s biodiversity index.

### Impacts on fisheries catch

**Fig 7:**
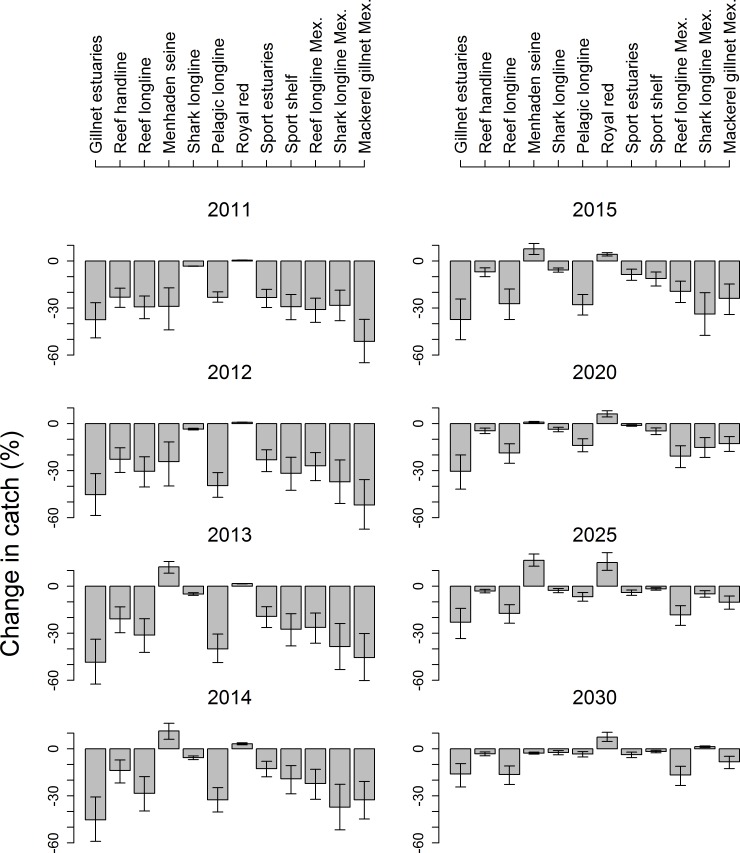
Projected annual catch for whole GOM. Catch presented relative to no-oil scenario. Error bars show range of sensitivity analysis; bars show mean.

The model predicts reduced catches the year after the oil spill (2011) in fleets targeting pelagic and reef-associated fish. Note that we have assumed no spatial reapportioning of effort or other adaptive changes by fishers. Generally, the model shows a 20–40% reduction in catch relative to the no oil scenario (Fig 7). Substantive losses are predicted in estuarine gillnet fisheries, mackerel fisheries and pelagic longline fisheries. Impacts on pelagic and some reef fisheries last into the 2020s according to the model, but there is a modest improvement in menhaden and royal red fisheries over that time. Fig 7 shows the predicted effect of the oil spill alone for comparability with the other results presented. S11 Fig indicates predicted losses of catch due to fishery closures.

### Comparison against ROV survey data

S12 Fig compares fish densities from ROV data with Atlantis estimates. The ROV data (W. Patterson, unpublished data) are influenced not only by changes in population size but also by fish movement between reefs. Nevertheless, there is qualitative agreement between the ROV data and model predictions in many cases. The model agrees with survey data that there was an initial decrease in fish numbers following the spill for all relevant functional groups except scamp and sciaenidae. The survey data shows post-spill fish density increases for six groups: large reef fish, shallow serranidae, small reef fish, jacks, other demersal fish and vermilion snapper. In all cases, the model agrees. The model also correctly predicts a lack of post-spill fish density increases in five groups: deep serranidae, red grouper, red snapper, greater amberjack and lutjanidae. However, in three groups, the model predicts post-spill fish density increases where none are seen in the observational data (gag grouper, scamp and sciaenidae). This suggests that unmodeled dynamics thwart recovery; notably, there are no recoveries in the ROV data that the model fails to predict. There are too few data available to assess predictions regarding large sharks or skates and rays. Thus, 11/16 functional groups show good agreement with data, capturing the initial decrease and subsequent increase in fish density (or lack thereof), 3/16 show marginal agreement (agreeing with the initial decrease or subsequent increase but not both), 2/16 cannot be assessed.

S13 Fig shows changes in body size predicted by the model relative to the ROV data. The model predicts realistic body sizes for jacks, lutjanidae, other demersal fish, red grouper, small reef fish and vermilion snapper indicating that the emergent body growth rates, which are a product mainly of consumption rates and mortality rates, are realistic. The model underestimates body sizes for gag grouper, red snapper, and scamp, though this result would be consistent with cases where fish present on the reefs are younger than the population average. The model is generally less variable than the observational data but this is expected since the model does not capture fish movement and averages body size data spatially and temporally. The model fails to predict an increase in the body size in red grouper and lutjanidae and a decrease in small reef fish. However, the quick rate of change in those data suggests this may partly be due to fish movement and not population-level change in weight at age. The model successfully predicts the increase in scamp body weight. Other groups, which do not show a clear directional change in the observations, are adequately represented by the model’s constant body size.

## Discussion

The model predicts that the DWH oil spill caused changes in biomass, age structure and distribution in a variety of fish guilds. In areas most heavily affected, the forage base lost a majority of its biomass. Although recovery for these high-turnover forage species is quick, the brief starvation period mounts on predator populations struggling to recover from toxicological impacts. Since demersal prey resources were affected more heavily than pelagic prey resources, there is more starvation in reef-associated and demersal predators then in pelagic predators. In general, there is heavier reliance on pelagic forage following the spill, although empirical data indicate the opposite pattern for red snapper [15]. This could increase predation risk for some demersal or reef-associated species forced to forage in pelagic areas. The shift towards pelagic forage holds implications for mitigation. A robust pelagic food web could help minimize starvation impacts among both pelagic and demersal predators. This is relevant for oil spills like the DWH with high rates of benthic deposition. It was also shown that food web effects have the potential to communicate biomass and fisheries losses far away from regions affected by oil. Injury assessment may consider that the area of impact is partly determined by the migratory range and/or connectivity of forage fish population.

Large pelagic fish were predicted to show the most severe, widespread and long-lasting impacts. In the model, this is because they transit the central GOM where most of the sub-surface plume was located. Fortunately, these projected impacts have not been realized in the catch record. One likely explanation is that avoidance behavior occurs at the mesoscale, allowing mobile fish to avoid patchy distributions of oil and thereby reduce exposure, such as has been demonstrated for sperm whales [43]. Oil avoidance behavior has also been documented for invertebrates and fish species in laboratory tank studies [44]. Such behavior in large pelagic fish may be key to understanding resiliency of pelagic food webs. Impacts on sedentary fish less able to move in response to toxicity may be more easily inferred by local oil concentrations. In this application, we find effects on territorial reef fish populations more predictable.

Changes in age structure may yet affect populations if initial losses in immature individuals translate to reduced breeding populations in years to come. Our study assumed a similar degree of physiological vulnerability in juvenile, sub-adult and adult fish. Despite this, young fish were more susceptible to the oil spill by virtue of their spatial distribution and food web dependencies. If juvenile fish are more sensitive to toxicological impacts than adults are, then age structure effects could be more pronounced than is shown here.

Intended only to protect consumers from contaminated seafood, fishery closures were too brief relative to the life cycle of many fish to promote recovery of stocks. The model indicates that the closures succeeded in reducing fisheries catch, but the reduction was not as large as suggested by McCrea-Strub et al. [45], who ignored the dynamic nature of the closures. With spatial reallocation of fishing effort (not represented here), the reduction in catch may have been still less for some species. The direct impacts of oil on larvae in the water column also had a relatively small and short-lived effect on fish populations compared to the loss of juvenile and adult fish. Although it was assumed that all larvae interacting with the oil were killed, the overlap in time and space between larval populations and the oil plume was small for most species relative to the total larvae released throughout the year [40]. A potentially larger and re-occurring effect on recruitment could result from the oiling of early life stage habitat, but this was not considered here.

In this study, we did not consider toxicological impacts on invertebrates or anoxia, but these factors may affect benthic food webs. Sedimentation of oil-associated marine snow has been demonstrated for the DWH oil spill [46] and impacts have been observed on meio- and macrofauna abundance and diversity [47,48]. Such impacts may occur perennially with resuspension by storms. Loss of benthic forage is therefore another factor that could potentially act on demersal and benthic fish populations, and fish populations that derive food indirectly from the benthic food web [49]. However, there are a number of assumptions required to extrapolate impacts from surveys to the large spatial domain of the Atlantis model. There is also not necessarily a monotonic relationship between oil exposure and benthic invertebrate abundance. There is some indication of a domed relationship between sedimentary oil load and invertebrate abundance (P. Montagna, Pers. Comm.) suggesting that enrichment, reduced predation pressure, or some other effect may be at play in addition to the toxic response [50].

Another factor not considered here is that oiled marine snow in the water column may provide a substrate on which increased rates of feeding and oil uptake can occur in agglomerated zooplankton, providing an express avenue for exposure in pelagic food webs. Hydrocarbon exposure and potentially exposure to dispersants may also cause genotoxicological impacts [51]. Multigenerational experiments are needed to inform this issue. Dispersants are toxic [52] and might increase the toxicity of oil, but a non-linear dose response means that lower oil concentrations could offset the increased toxicity. We have not explicitly modeled dispersants here but validation against ROV data suggests that we are close in our approximation of the combined toxicological effects of oil and dispersant. We did not consider the effect of chronic toxicity on fish, e.g., from resuspension of contaminated sediments or repeated spills, but chronic effects could manifest as sub-lethal population-level impacts [53].

The findings of this study are sensitive to model assumptions, such as those concerning relationships between groups as defined by the diet matrix. However, sensitivity analysis in Atlantis is hampered by the model’s long run time and large number of parameters [16,17]. Morzaria et al. [54] explore the error around the [K1000 *β*363] simulation tested here using a new statistical methodology and parallel computing.

## Conclusions

The scale and depth of the DWH oil spill distinguish it from previous oil spills in the United States but this experience may serve as the prototype for future spills given that ultradeep petroleum exploration and extraction is becoming more common. Availability of pelagic forage could be important in factor in determining ecosystem resiliency, particularly in the case of oil spills like the DWH that have heavy deposition and large impacts on benthic forage. Injury assessment needs to consider a wider area of impact than the footprint of the oil spill and long timescales. The model results suggest that recovery of high-turnover populations generally happens within 10 years, but slow-growing populations might take 30+ years to recover. We should be mindful of delayed impacts to fisheries caused by shifts in age distribution. The potential window of effect will correspond to the age at maturity of exploited species.

## Supporting information

S1 Fig Sediment PAH vs water column PAH. Sediment PAH concentration measured in C-IMAGE sediment sampling (Romero and Hollander, unpublished data) versus time- and depth- integrated water column PAH concentrations from the Coastal Modeling System. Dotted lines show sediment:water column ratios for reference.
(PDF)

S2 Fig Relative biomass changes caused by fishery closures and recruitment impacts. Fishery closures (top); recruitment impacts (bottom); no oil effects are incorporated.
(PDF)

S3 Fig Commercial catch of large pelagic fish. Catch is shown for species constituting the large pelagic guild before the oil spill (dark grey bars: average of 2007–2010) and after the oil spill (light grey bars: average of 2010–2014). Source: ICCAT and NMFS.
(PDF)

S4 Fig Age composition changes. Differences in age composition between no-oil (dark gray) and oiled (light grey) scenarios. Condition is shown for October 2010 for a subset of polygons that experienced the greatest oil impacts. Represents oil simulation [K1000 *β*363]. Relative proportion is shown.
(PDF)

S5 Fig Biomass of immature and mature cohorts before and after spill. Biomass in immature age classes and mature age classes for species assessed by SEDAR since 2012. Pre-spill shows average of 2009 and 2010, post-spill shows average of 2011 and 2012. The immature/mature age division is consistent with the juvenile/adult division used in Atlantis. References and notes provided in S3 Table.
(PDF)

S6 Fig Mature-to-immature numbers ratio. No-oil scenario (dotted line); oiled scenario (solid line) Represents oil simulation [K1000 *β*363].
(PDF)

S7 Fig Absolute biomass reduction for no oil versus oil scenario. Biomass minima is shown occurring 7–16 months (median 10 months) after the oil spill. The areas of major impact typically occur on the continental shelves far from the oil plume, where concentrations of the affected populations occur. Represents oil simulation [K1000 *β*363].
(PDF)

S8 Fig Per capita consumption rate on prey by guild. Area of circle is proportional to the per capita consumption rate. Both predator and prey are presented at aggregated guild level. Only prey items constituting >1% of the diet are presented. Large demersal fish (LDF), Sciaendiae (SCI), Elasmobranchs (ELA), Large pelagic fish (LPF), Groupers (GRP), Snappers (SNP), Small demersal and reef fish (SDR), Small pelagic fish (SPL), other prey items (OTH). No oil scenario and oiled scenario both show day 300 of the simulation (Oct 28, 2010) when biomass impacts were pronounced.
(PDF)

S9 Fig Condition factor of fish represented as reserve:structural nitrogen ratio. Reserve represents soft body tissue that can be reabsorbed (e.g. muscle, fat, gonads), structural represents hard tissues and structures (e.g., bone). High rN/sN indicates good body condition. Dotted line: no oil scenario, solid line: oiled [K1000 *β*363]. Seasonal saw-toothed pattern (present in both scenarios) reflects gonadal tissue loss in spawning.
(PDF)

S10 Fig Ecosystem indicators for no-oil and oiled scenario. Oil (dotted line); no-oil (solid line). Mean trophic level of ecosystem, pelagic-to-demersal biomass ratio for fish, piscivorous-to-planktivorous biomass ratio for fish, and Shannon biodiversity. Represents oil simulation [K1000 *β*363].
(PDF)

S11 Fig Change in catch for the entire GOM due to fishery closures. No oil effects are included.
(PDF)

S12 Fig Comparison of fish count density data from remotely operated vehicles with numbers of fish from Atlantis. ROV data have been aggregated by species into Atlantis functional groups. Gray circles show densities measured at each site and sampling date (median value: dotted lines). The Atlantis numbers (solid lines) have been scaled so that median matches ROV data.
(PDF)

S13 Fig Fish body size predicted from the model versus ROV reef surveys. Model (red dotted line); laser-scaled fish size estimates from ROV surveys (black line: median; bars: lower and upper quartiles, whiskers: ±2*interquartile range, dots: outliers). ROV data have been converted to individual body weight using a length-weight relationship.
(PDF)

S1 Table Composition of guilds and functional group-level data. Recovery to 99% of pre-spill biomass. See Ainsworth et al. [17] for species memberships in Atlantis functional groups. Note that the Atlantis model contains 91 functional groups in total. DNR: Did not recover. Represents oil simulation [K1000 *β*363].
(PDF)

S2 Table Sensitivity analysis of biomass. Shows smallest observed biomass for various guilds relative to no-oil scenario. Biomasses are summed across all functional groups within these guilds. Biomass minima occur 7–16 months (median 10 months) after the oil spill. Parameters varied are sediment:water column concentration factor (K) and threshold for oil impacts (*β*). Red and blue cells represent greatest and least potential impact, respectively.
(PDF)

S3 Table References for SEDAR age structure data. (PDF)
