# Supplementary material for: Impacts of the Deepwater Horizon oil spill evaluated using an end-to-end ecosystem model
Source: PLoS One. 2018 Jan 25;13(1):e0190840. doi: 10.1371/journal.pone.0190840 (PMC5784916; doi:10.1371/journal.pone.0190840)
Supplement: S1 Fig — Sediment PAH concentration measured in C-IMAGE sediment sampling (Romero and Hollander, unpublished data) versus time- and depth- integrated water column PAH concentrations from the Coastal Modeling System. Dotted lines show sediment:water column ratios for reference. (PDF) [file pone.0190840.s001.pdf]

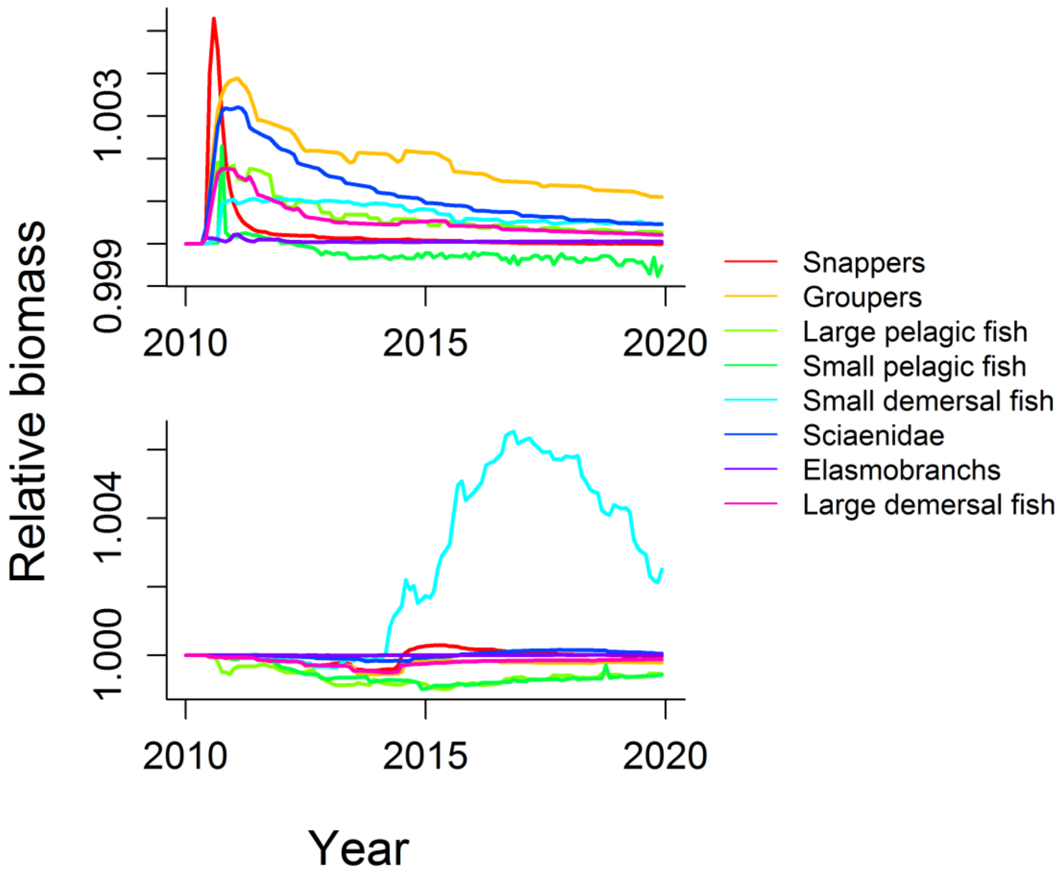

S1 Fig. Relative biomass in oiled [scenario K1000 β373] vs. non-oiled scenarios caused by fishery closures (top) and recruitment impacts (bottom).
