# Supplementary material for: Impacts of the Deepwater Horizon oil spill evaluated using an end-to-end ecosystem model
Source: PLoS One. 2018 Jan 25;13(1):e0190840. doi: 10.1371/journal.pone.0190840 (PMC5784916; doi:10.1371/journal.pone.0190840)
Supplement: S2 Fig — Fishery closures (top); recruitment impacts (bottom); no oil effects are incorporated. (PDF) [file pone.0190840.s002.pdf]

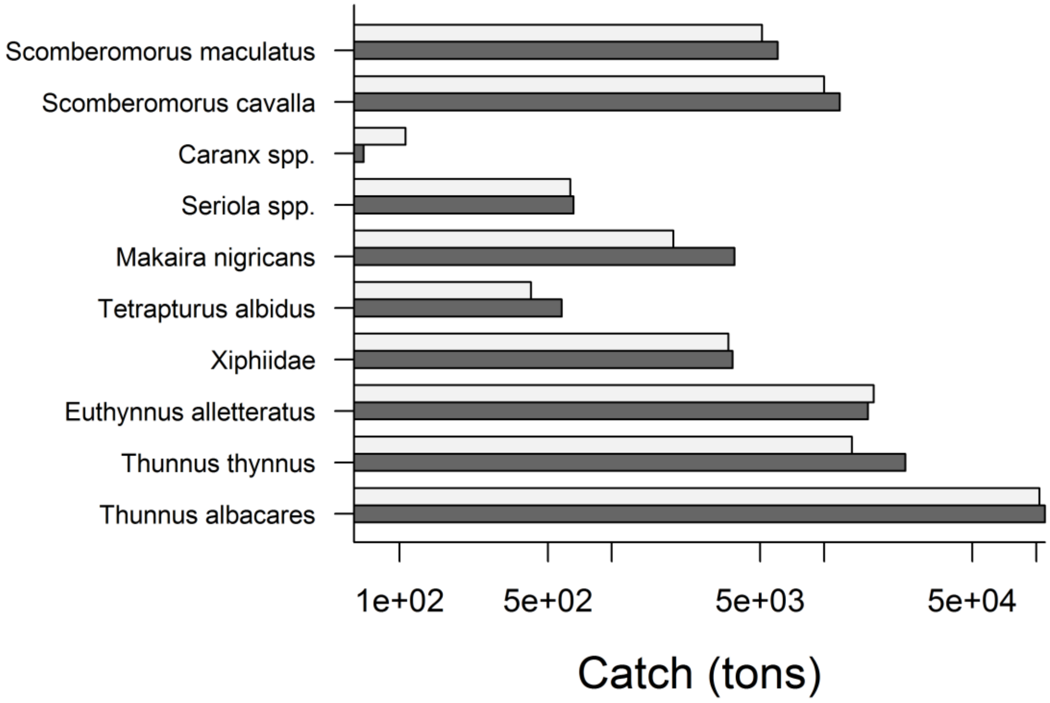

S2 Fig. Commercial catch of large pelagic species constituting the large pelagic guild before the oil spill (dark grey bars: average of 2007-2010) and after the oil spill (light grey bars: average of 2010-2014). Source: ICCAT and NMFS.
