# Supplementary material for: Impacts of the Deepwater Horizon oil spill evaluated using an end-to-end ecosystem model
Source: PLoS One. 2018 Jan 25;13(1):e0190840. doi: 10.1371/journal.pone.0190840 (PMC5784916; doi:10.1371/journal.pone.0190840)
Supplement: S3 Fig — Catch is shown for species constituting the large pelagic guild before the oil spill (dark grey bars: average of 2007–2010) and after the oil spill (light grey bars: average of 2010–2014). Source: ICCAT and NMFS. (PDF) [file pone.0190840.s003.pdf]

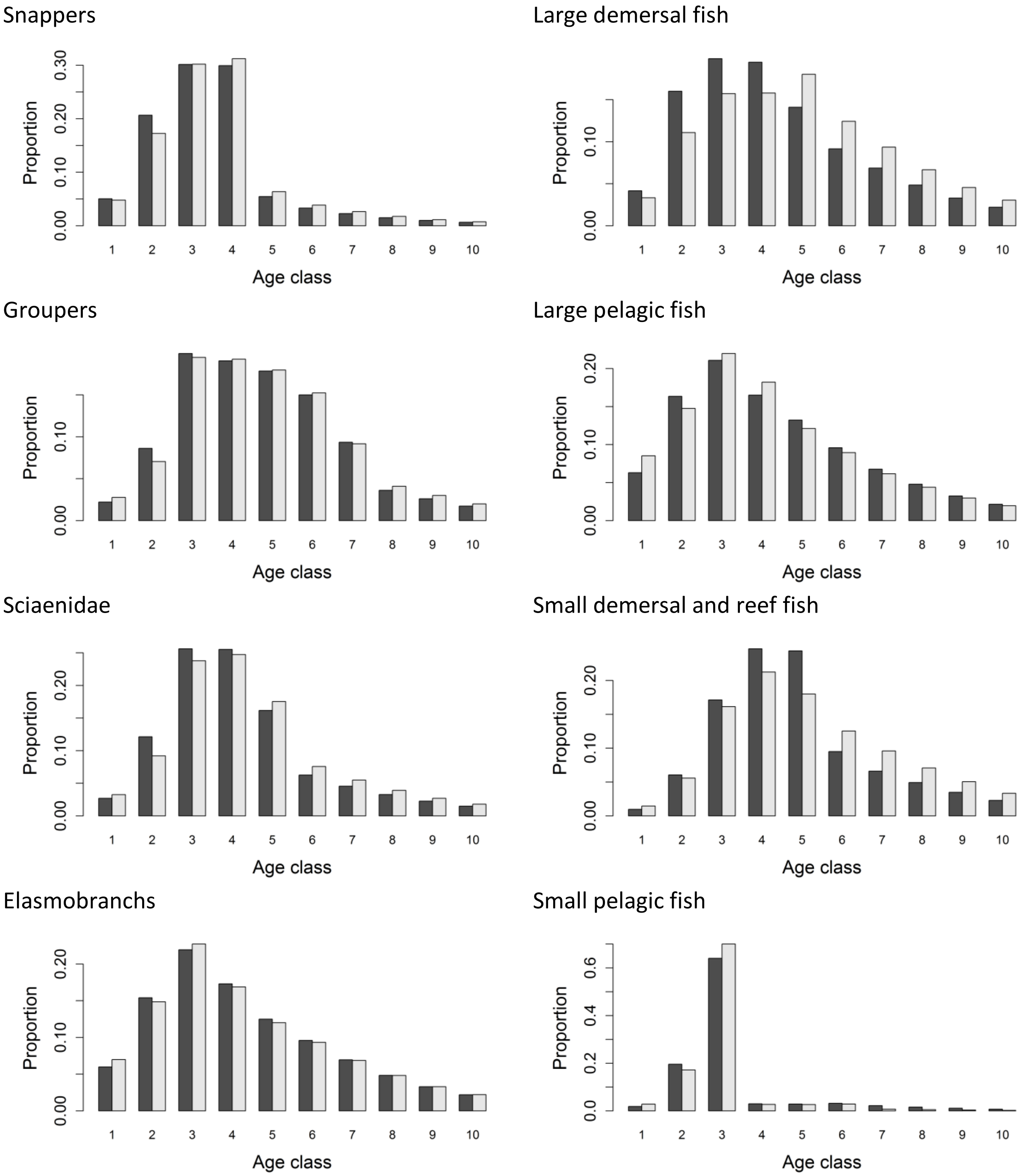

S3 Fig. Differences in age composition between no-oil (dark gray) and oiled (light grey) scenarios. Condition is shown for October 2010 for a subset of polygons that experienced the greatest oil impacts. Represents oil simulation [K1000 β363]. Relative proportion is shown.
