# Supplementary material for: Impacts of the Deepwater Horizon oil spill evaluated using an end-to-end ecosystem model
Source: PLoS One. 2018 Jan 25;13(1):e0190840. doi: 10.1371/journal.pone.0190840 (PMC5784916; doi:10.1371/journal.pone.0190840)
Supplement: S4 Fig — Differences in age composition between no-oil (dark gray) and oiled (light grey) scenarios. Condition is shown for October 2010 for a subset of polygons that experienced the greatest oil impacts. Represents oil simulation [K1000 β363]. Relative proportion is shown. (PDF) [file pone.0190840.s004.pdf]

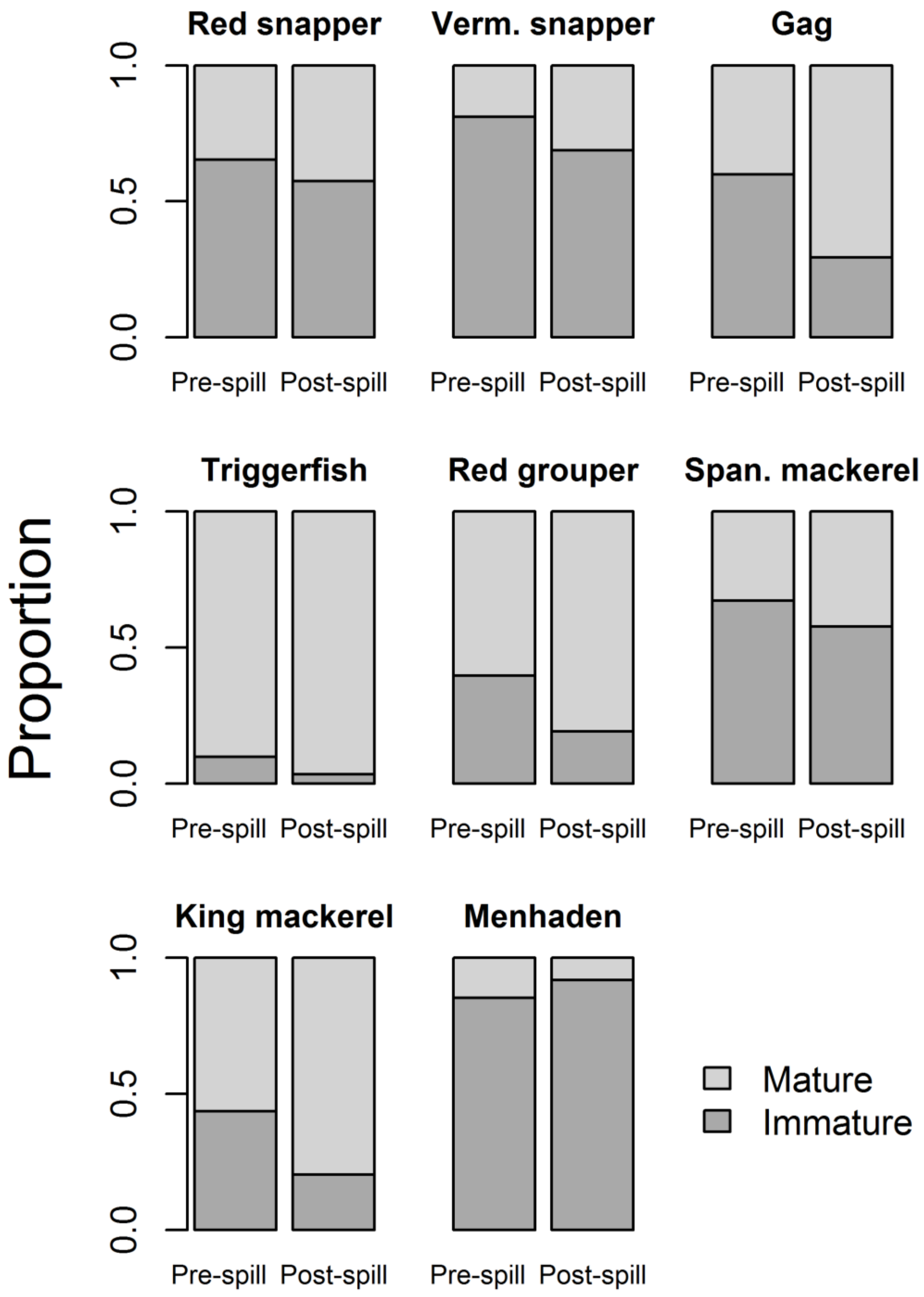

S4 Fig. Biomass in immature age classes and mature age classes for species assessed by SEDAR since 2012. Pre-spill shows average of 2009 and 2010, post-spill shows average of 2011 and 2012. The immature/mature age division is consistent with the juvenile/adult division used in Atlantis (Ainsworth et al. 2015). References and notes provided in Appendix Table A3.
