# Supplementary material for: Impacts of the Deepwater Horizon oil spill evaluated using an end-to-end ecosystem model
Source: PLoS One. 2018 Jan 25;13(1):e0190840. doi: 10.1371/journal.pone.0190840 (PMC5784916; doi:10.1371/journal.pone.0190840)
Supplement: S5 Fig — Biomass in immature age classes and mature age classes for species assessed by SEDAR since 2012. Pre-spill shows average of 2009 and 2010, post-spill shows average of 2011 and 2012. The immature/mature age division is consistent with the juvenile/adult division used in Atlantis. References and notes provided in S3 Table. (PDF) [file pone.0190840.s005.pdf]

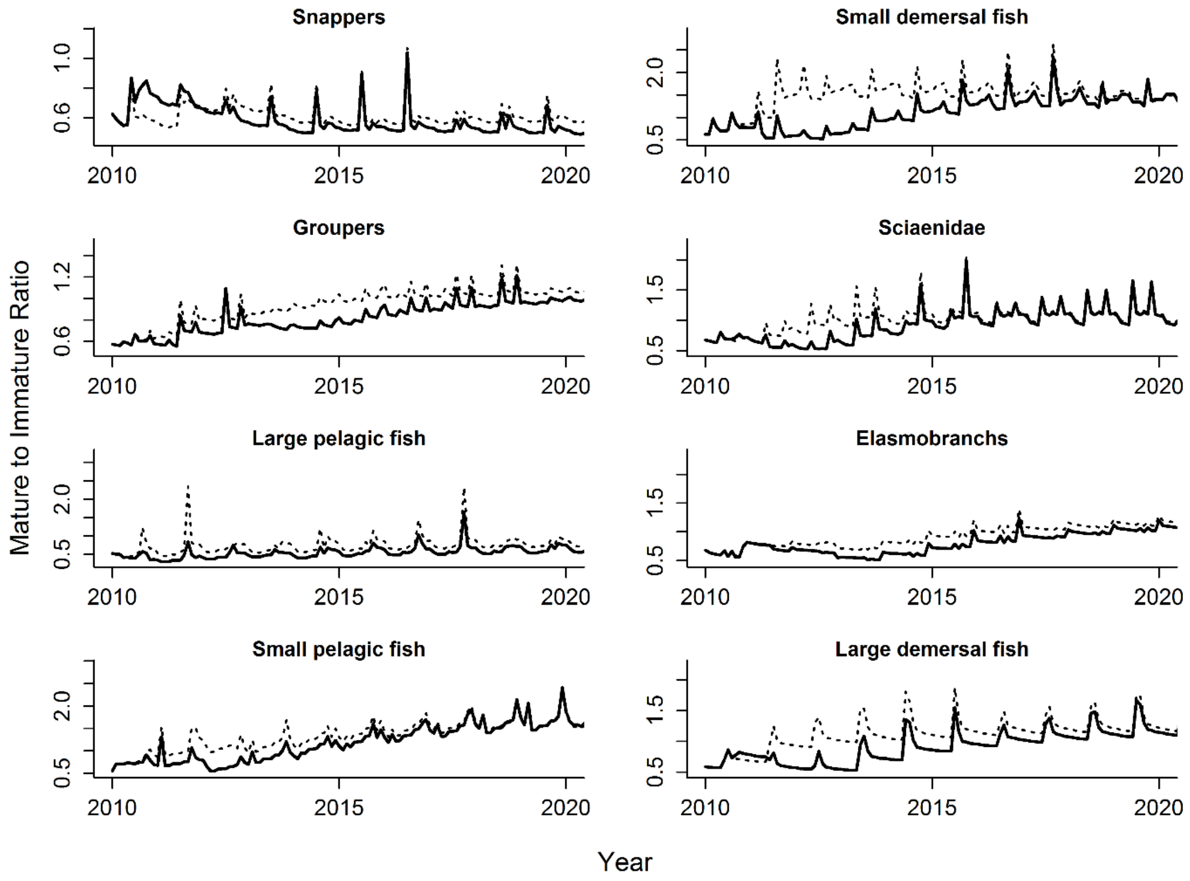

S5 Fig. Mature-to-immature numbers ratio for no-oil scenario (dotted line) and oiled scenario [K1000 β363] (solid line).
