# Supplementary material for: Impacts of the Deepwater Horizon oil spill evaluated using an end-to-end ecosystem model
Source: PLoS One. 2018 Jan 25;13(1):e0190840. doi: 10.1371/journal.pone.0190840 (PMC5784916; doi:10.1371/journal.pone.0190840)
Supplement: S6 Fig — No-oil scenario (dotted line); oiled scenario (solid line) Represents oil simulation [K1000 β363]. (PDF) [file pone.0190840.s006.pdf]

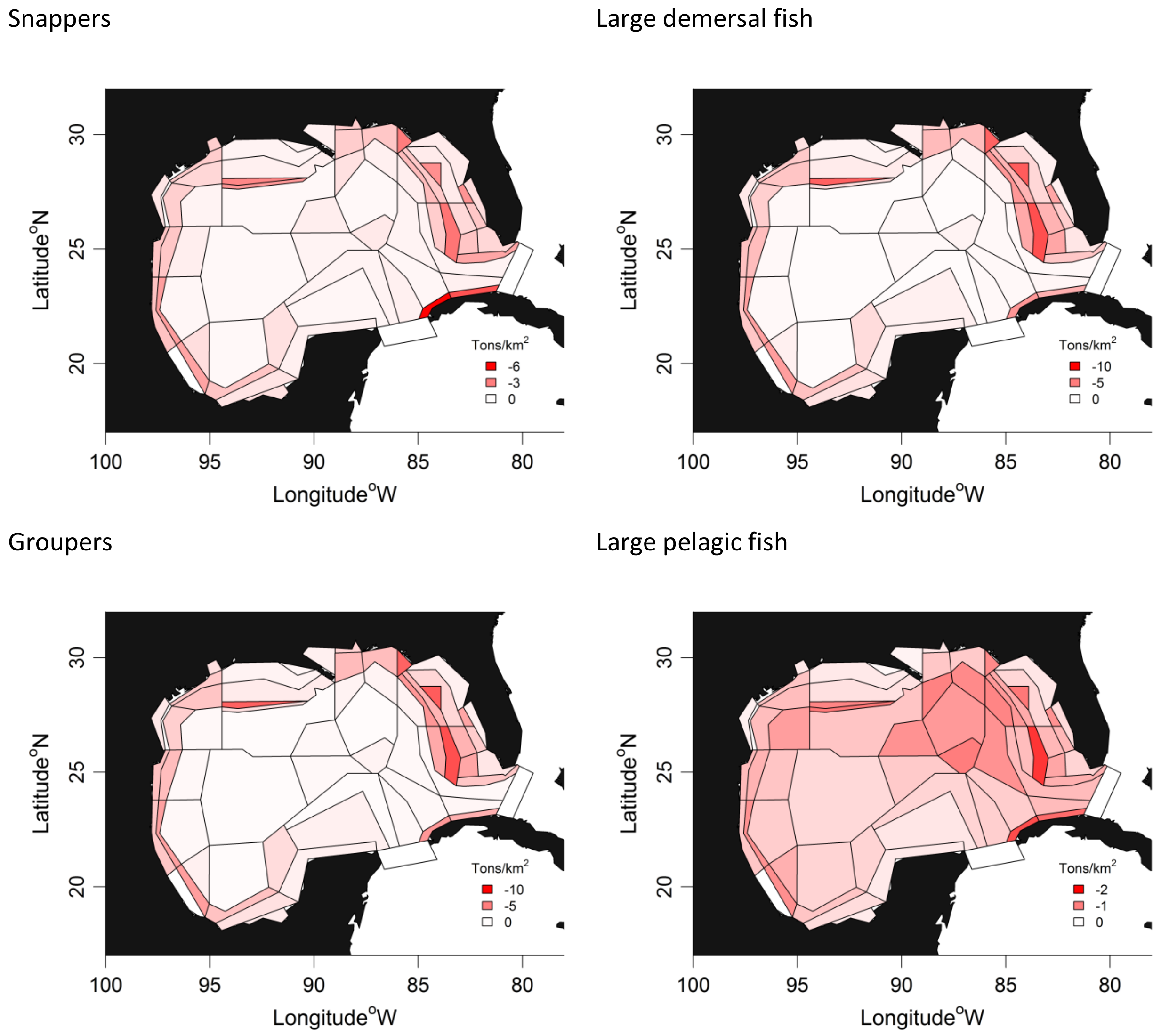

S6 Fig. Absolute biomass reduction for no oil versus oil scenario. Biomass minima is shown occurring 7-16 months (median 10 months) after the oil spill. Oil simulation [K1000 β363].

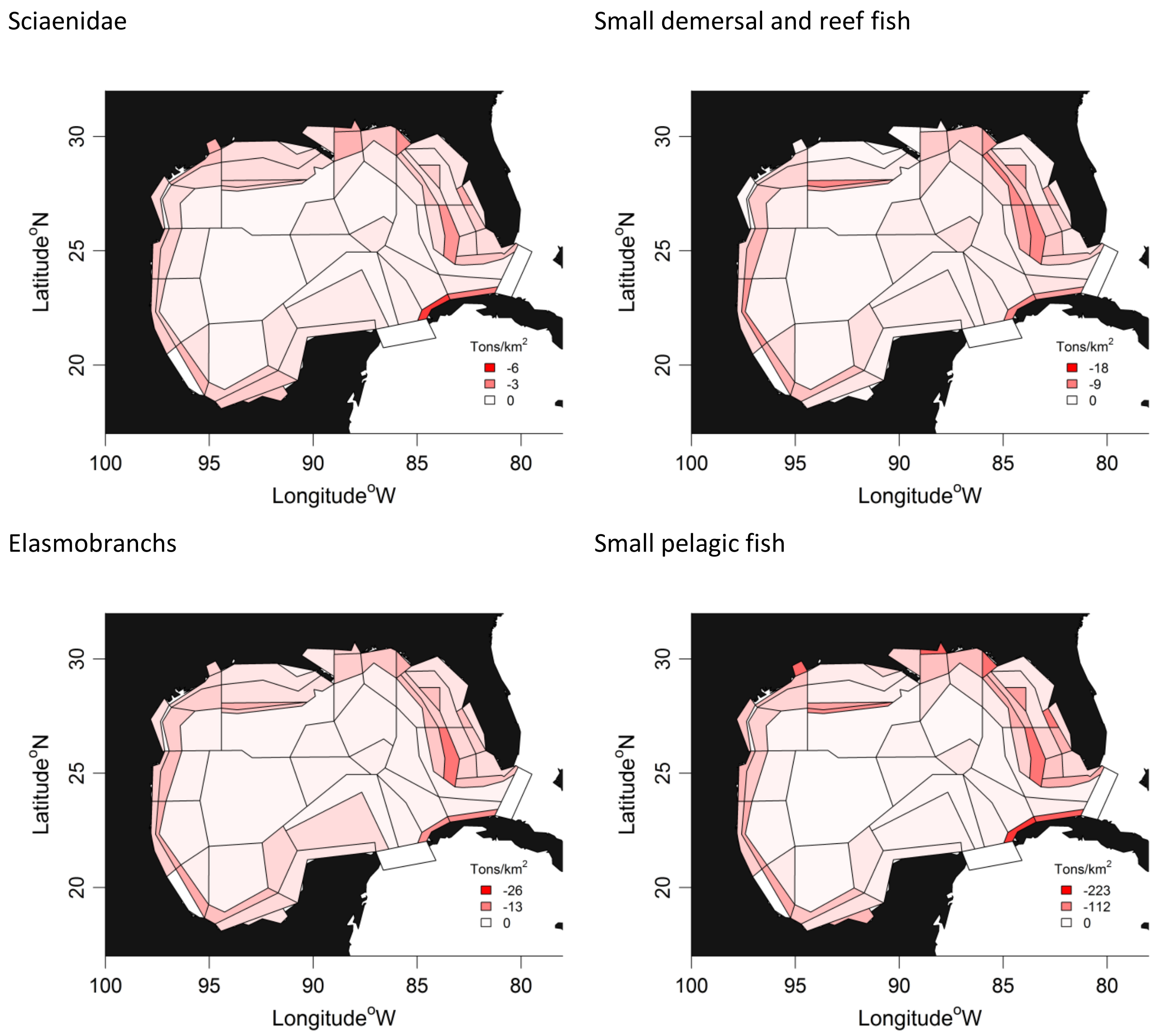
Sciaenidae
Small demersal and reef fish
Elasmobranchs
Small pelagic fish
Latitude°N
Longitude°W
30
25
20
100
95
90
85
80
Tons/km²
-6
-3
0
-18
-9
0
-26
-13
0
-223
-112
0

S6 Fig (cont.).
