# Supplementary material for: Impacts of the Deepwater Horizon oil spill evaluated using an end-to-end ecosystem model
Source: PLoS One. 2018 Jan 25;13(1):e0190840. doi: 10.1371/journal.pone.0190840 (PMC5784916; doi:10.1371/journal.pone.0190840)
Supplement: S7 Fig — Biomass minima is shown occurring 7–16 months (median 10 months) after the oil spill. The areas of major impact typically occur on the continental shelves far from the oil plume, where concentrations of the affected populations occur. Represents oil simulation [K1000 β363]. (PDF) [file pone.0190840.s007.pdf]

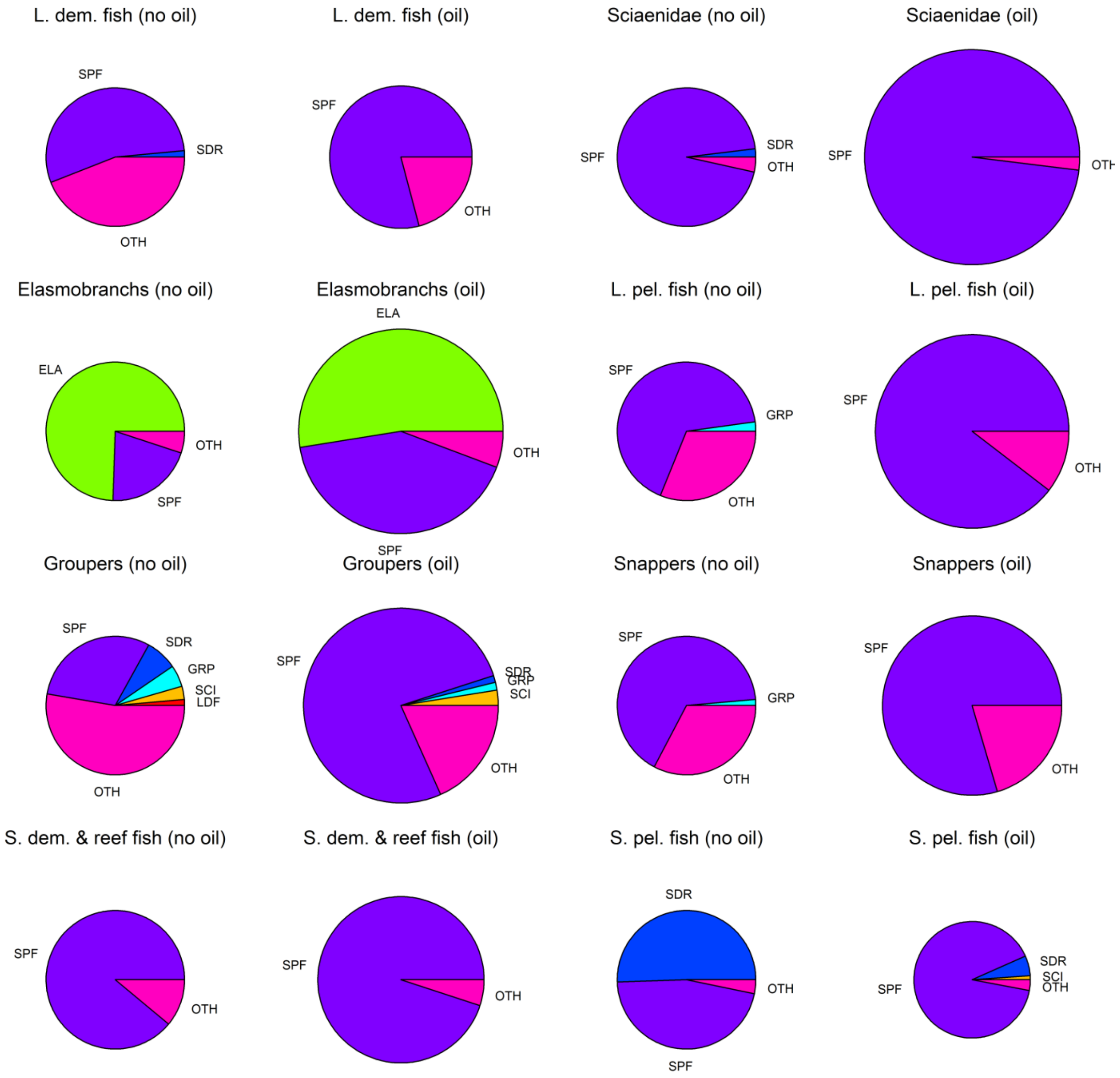

S7 Fig. Per capita consumption rate on prey by guild. Area of circle is proportional to the per capita consumption rate. Both predator and prey are presented at aggregated guild level. Only prey items constituting >1% of the diet are presented. Large demersal fish (LPF), Sciaendiae (SCI), Elasmobranchs (ELA), Large pelagic fish (LPF), Groupers (GRP), Snappers (SNP), Small demersal and reef fish (SDR), Small pelagic fish (SPL), other prey items (OTH). No oil scenario and oiled scenario both show day 300 of the simulation, near the peak of the oil spill.
