# Supplementary material for: Impacts of the Deepwater Horizon oil spill evaluated using an end-to-end ecosystem model
Source: PLoS One. 2018 Jan 25;13(1):e0190840. doi: 10.1371/journal.pone.0190840 (PMC5784916; doi:10.1371/journal.pone.0190840)
Supplement: S8 Fig — Area of circle is proportional to the per capita consumption rate. Both predator and prey are presented at aggregated guild level. Only prey items constituting >1% of the diet are presented. Large demersal fish (LDF), Sciaendiae (SCI), Elasmobranchs (ELA), Large pelagic fish (LPF), Groupers (GRP), Snappers (SNP), Small demersal and reef fish (SDR), Small pelagic fish (SPL), other prey items (OTH). No oil scenario and oiled scenario both show day 300 of the simulation (Oct 28, 2010) when biomass impacts were pronounced. (PDF) [file pone.0190840.s008.pdf]

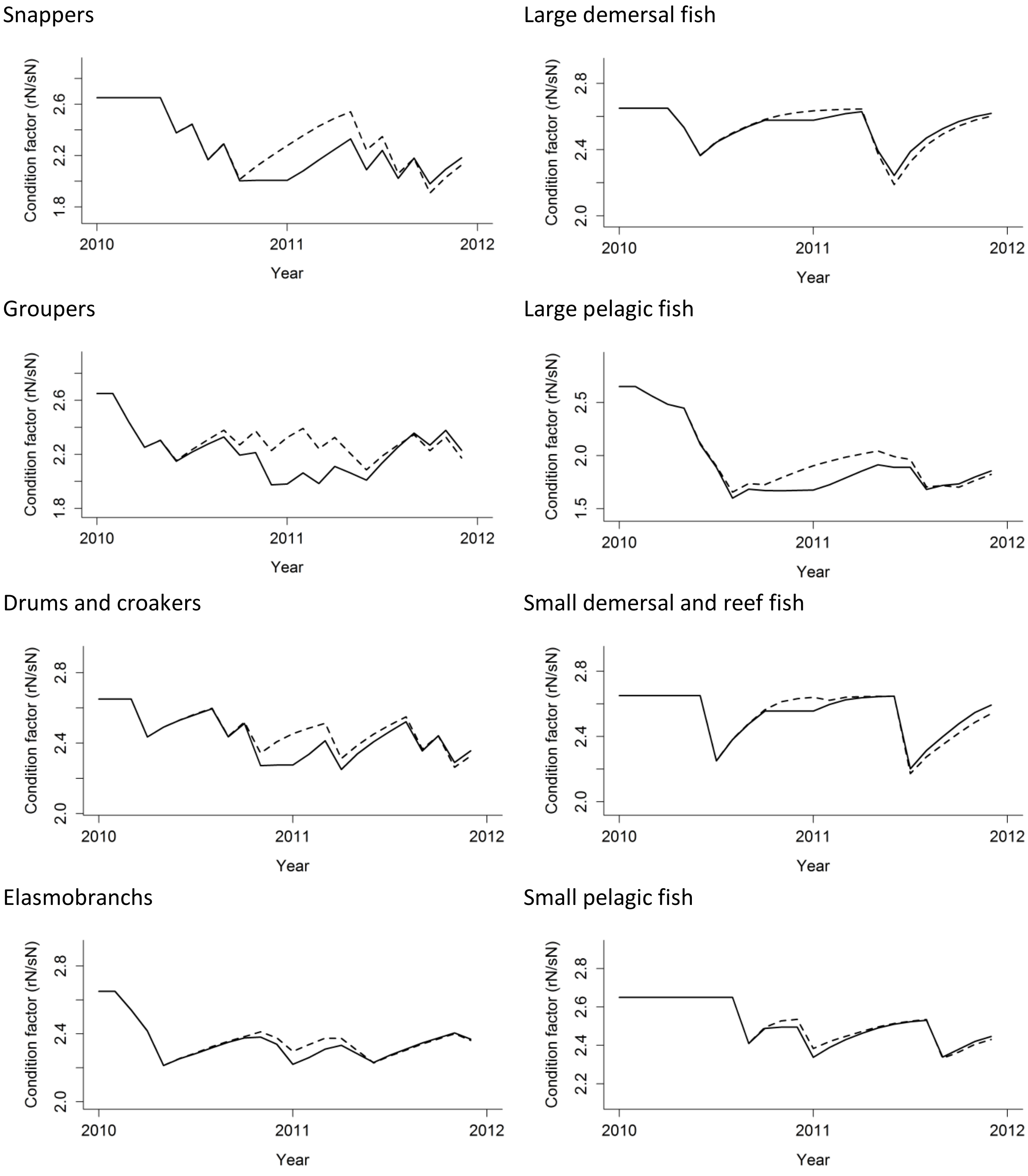

S8 Fig. Condition factor of fish represented as reserve:structural Nitrogen ratio. Reserve represents soft body tissue that can be reabsorbed (e.g. muscle, fat, gonads), structural represents hard tissues and structures (e.g., bone). High rN/sN indicates good body condition. Dotted line: no oil scenario, solid line: oiled [K1000 β363]. Seasonal saw-toothed pattern (present in both scenarios) reflects gonadal tissue loss in spawning.
