# Supplementary material for: Impacts of the Deepwater Horizon oil spill evaluated using an end-to-end ecosystem model
Source: PLoS One. 2018 Jan 25;13(1):e0190840. doi: 10.1371/journal.pone.0190840 (PMC5784916; doi:10.1371/journal.pone.0190840)
Supplement: S9 Fig — Reserve represents soft body tissue that can be reabsorbed (e.g. muscle, fat, gonads), structural represents hard tissues and structures (e.g., bone). High rN/sN indicates good body condition. Dotted line: no oil scenario, solid line: oiled [K1000 β363]. Seasonal saw-toothed pattern (present in both scenarios) reflects gonadal tissue loss in spawning. (PDF) [file pone.0190840.s009.pdf]

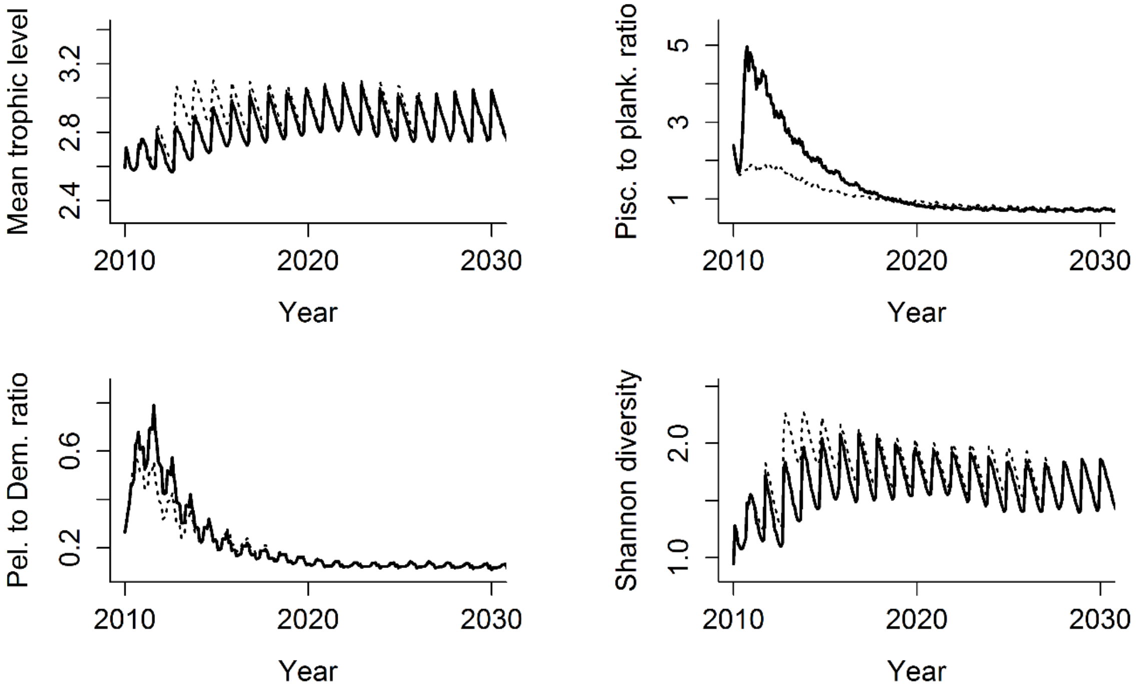

S9 Fig. Ecosystem indicators for the no-oil scenario (dotted line) and oiled scenario [K1000 β363] (solid line). Mean trophic level of ecosystem, pelagic-to-demersal biomass ratio for fish, piscivorous-to-planktivorous biomass ratio for fish, and Shannon biodiversity.
