# Supplementary material for: Impacts of the Deepwater Horizon oil spill evaluated using an end-to-end ecosystem model
Source: PLoS One. 2018 Jan 25;13(1):e0190840. doi: 10.1371/journal.pone.0190840 (PMC5784916; doi:10.1371/journal.pone.0190840)
Supplement: S10 Fig — Oil (dotted line); no-oil (solid line). Mean trophic level of ecosystem, pelagic-to-demersal biomass ratio for fish, piscivorous-to-planktivorous biomass ratio for fish, and Shannon biodiversity. Represents oil simulation [K1000 β363]. (PDF) [file pone.0190840.s010.pdf]

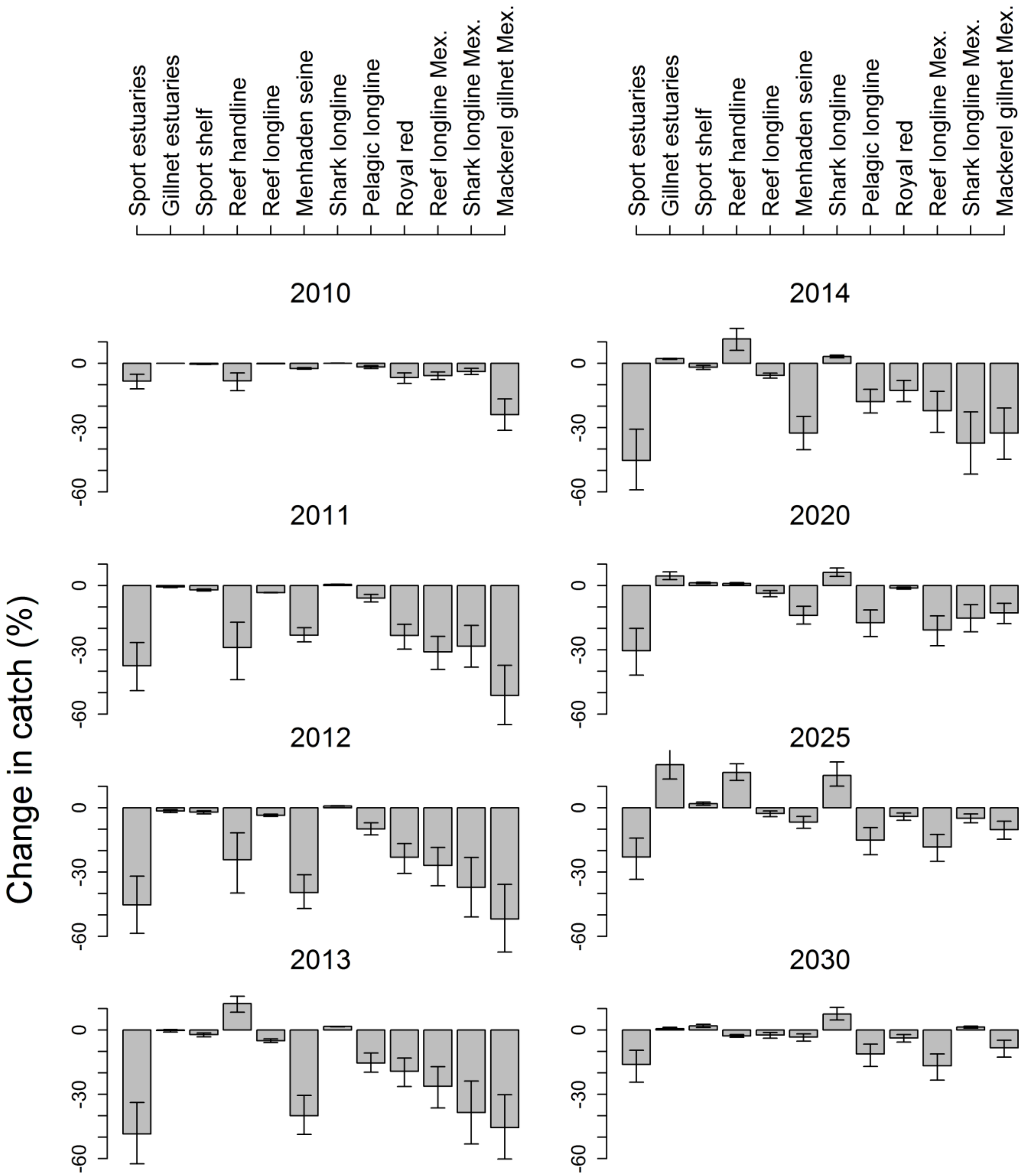

S10 Fig. Projected annual catch by fleet in oil scenario versus no-oil scenario. Error bars show range of sensitivity analysis; bars show mean.
