# Supplementary material for: Impacts of the Deepwater Horizon oil spill evaluated using an end-to-end ecosystem model
Source: PLoS One. 2018 Jan 25;13(1):e0190840. doi: 10.1371/journal.pone.0190840 (PMC5784916; doi:10.1371/journal.pone.0190840)
Supplement: S11 Fig — No oil effects are included. (PDF) [file pone.0190840.s011.pdf]

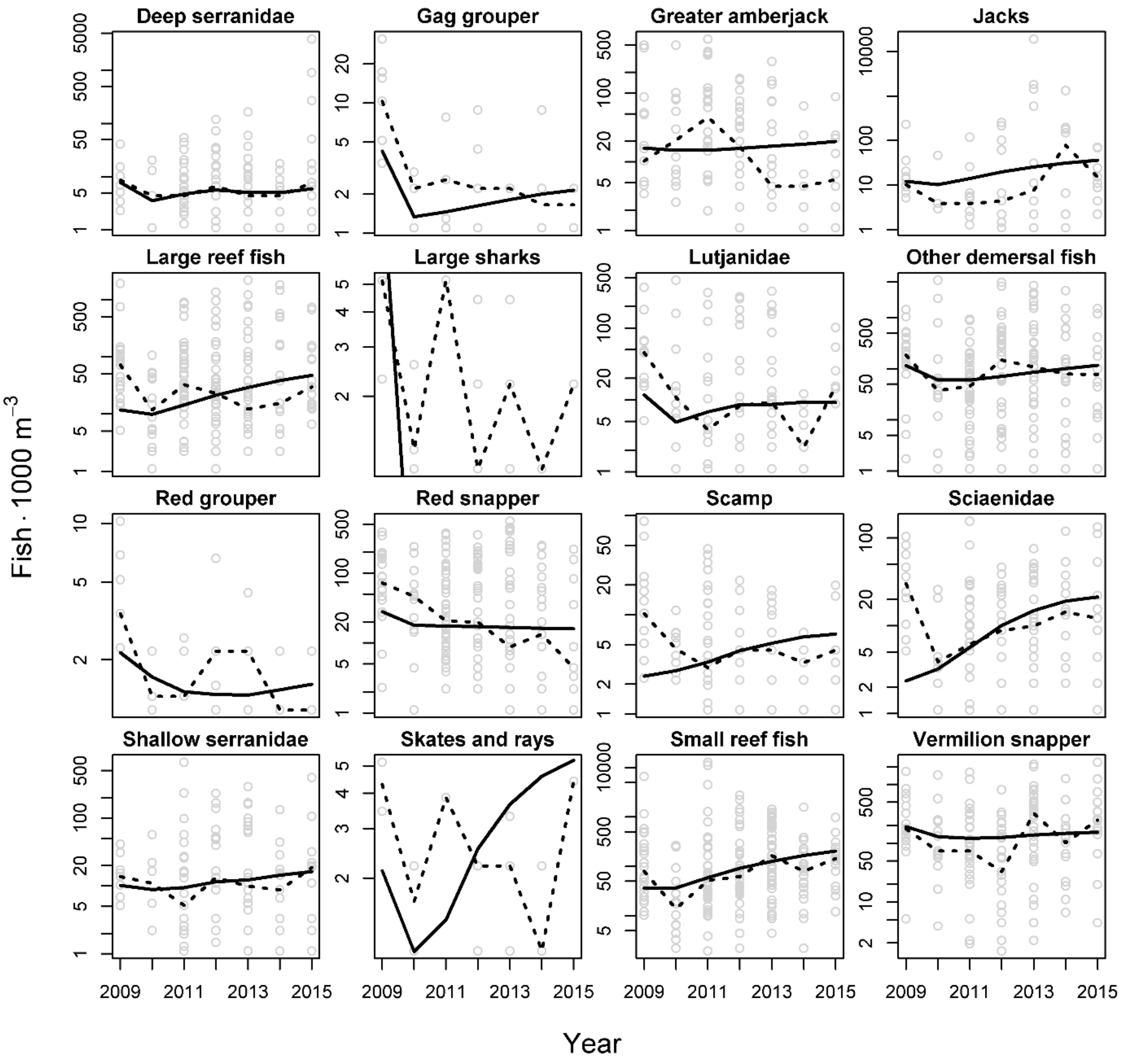

S11 Fig. Comparison of fish count density data from remotely operated vehicles with numbers of fish from Atlantis (polygon 39). ROV data have been aggregated by species into Atlantis functional groups. Gray circles show densities measured at each site and sampling date (median value: dotted lines). The Atlantis numbers (solid lines) have been scaled so that median matches ROV data.
