# Supplementary material for: Impacts of the Deepwater Horizon oil spill evaluated using an end-to-end ecosystem model
Source: PLoS One. 2018 Jan 25;13(1):e0190840. doi: 10.1371/journal.pone.0190840 (PMC5784916; doi:10.1371/journal.pone.0190840)
Supplement: S12 Fig — ROV data have been aggregated by species into Atlantis functional groups. Gray circles show densities measured at each site and sampling date (median value: dotted lines). The Atlantis numbers (solid lines) have been scaled so that median matches ROV data. (PDF) [file pone.0190840.s012.pdf]

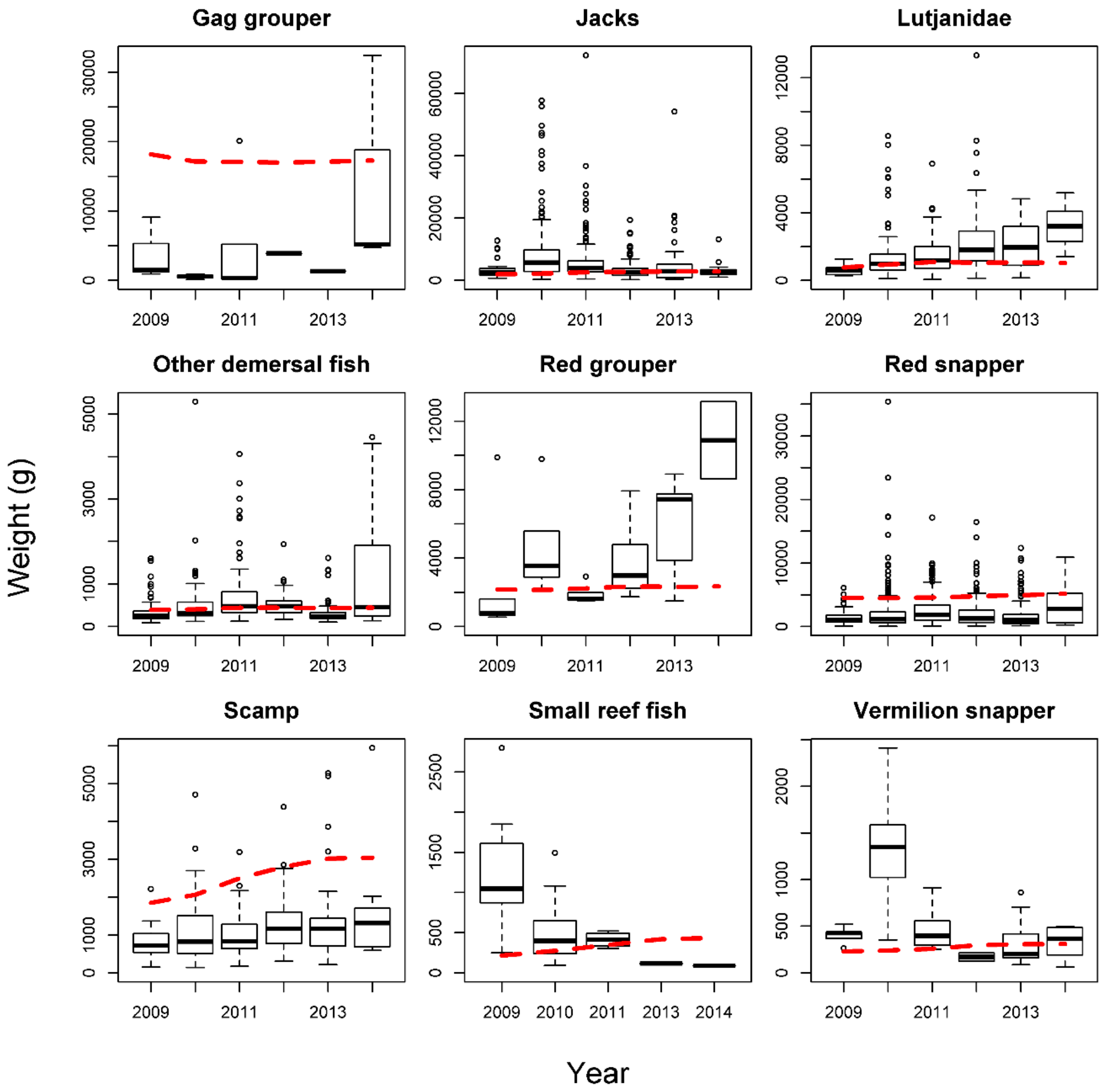

S12 Fig. Fish body size predicted from the model in polygon 39 (red dotted line) versus laser-scaled fish size estimates from remotely operated vehicle reef surveys (black line: median; bars: lower and upper quartiles, whiskers: ±2*interquartile range, dots: outliers). ROV data have been converted to individual body weight using a length-weight relationship.
