# Supplementary material for: Impacts of the Deepwater Horizon oil spill evaluated using an end-to-end ecosystem model
Source: PLoS One. 2018 Jan 25;13(1):e0190840. doi: 10.1371/journal.pone.0190840 (PMC5784916; doi:10.1371/journal.pone.0190840)
Supplement: S13 Fig — Model (red dotted line); laser-scaled fish size estimates from ROV surveys (black line: median; bars: lower and upper quartiles, whiskers: ±2*interquartile range, dots: outliers). ROV data have been converted to individual body weight using a length-weight relationship. (PDF) [file pone.0190840.s013.pdf]

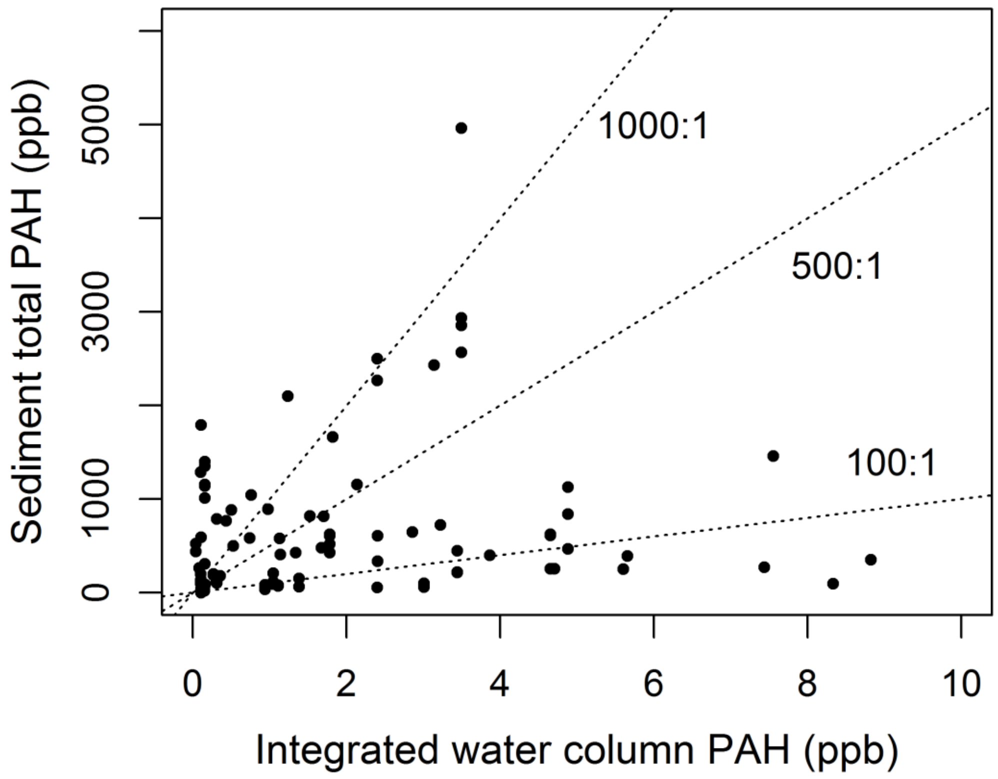

S13 Fig. Sediment PAH concentration measured in C-IMAGE sediment sampling (Romero and Hollander, unpublished data) versus time- and depth- integrated water column PAH concentrations from the Coastal Modeling System (Paris et al. 2012). Dotted lines show sediment:water column ratios for reference.
