# Supplementary material for: Impacts of the Deepwater Horizon oil spill evaluated using an end-to-end ecosystem model
Source: PLoS One. 2018 Jan 25;13(1):e0190840. doi: 10.1371/journal.pone.0190840 (PMC5784916; doi:10.1371/journal.pone.0190840)
Supplement: S1 Table — Recovery to 99% of pre-spill biomass. See Ainsworth et al. [17] for species memberships in Atlantis functional groups. Note that the Atlantis model contains 91 functional groups in total. DNR: Did not recover. Represents oil simulation [K1000 β363]. (PDF) [file pone.0190840.s014.pdf]

	Guild									
Atlantis functional group	Snappers	Groupers	Large pelagic fish	Small pelagic fish	Small demersal & reef fish	Sciaenidae	Elasmobranchs	Large demersal fish	Recovery time (years)	Biomass minimum vs. no-oil
Gag grouper		■							18	0.3
Red grouper		■							10	0.68
Scamp		■							18	0.67
Shallow serranidae		■							6	0.54
Deep serranidae		■							DNR	0.25
Red snapper	■								20	0.61
Vermilion snapper	■								8	0.65
Lutjanidae	■								4	0.56
Small reef fish					■				9	0.32
Black drum						■			6	0.31
Red drum						■			13	0.71
Sciaenidae						■			5	0.45
Flatfish								■	9	0.32
Other demersal fish								■	21	0.35
Small demersal fish					■				4	0.13
Yellowfin tuna			■						24	0.18
Bluefin tuna			■						47	0.3
Little tunny			■						17	0.13
Other tuna			■						30	0.06
Swordfish			■						34	0.01
White marlin			■						DNR	0.22
Blue marlin			■						44	0.25
Other billfish			■						DNR	0.32
Greater amberjack			■						47	0.69
Jacks			■						10	0.5
King mackerel			■						11	0.83
Spanish mackerel			■						4	0.41
Spanish sardine				■					19	0.63
Menhaden				■					4	0.4
Pinfish				■					2	0.82
Small pelagic fish				■					9	0.2
Blacktip shark							■		4	0.64
Benthic feeding sharks							■		17	0.31
Large sharks							■		18	0.9
Filter feeding sharks							■		40	0.35
Small sharks							■		14	0.78
Skates and rays							■		8	0.32

S1 Table. Composition of guilds and functional group-level data. See Ainsworth et al. (2015) for species memberships in Atlantis functional groups. Recovery to 99% of pre-spill biomass [scenario K1000 β373]. DNR: Did not recover.
