# Supplementary material for: Impacts of the Deepwater Horizon oil spill evaluated using an end-to-end ecosystem model
Source: PLoS One. 2018 Jan 25;13(1):e0190840. doi: 10.1371/journal.pone.0190840 (PMC5784916; doi:10.1371/journal.pone.0190840)
Supplement: S2 Table — Shows smallest observed biomass for various guilds relative to no-oil scenario. Biomasses are summed across all functional groups within these guilds. Biomass minima occur 7–16 months (median 10 months) after the oil spill. Parameters varied are sediment:water column concentration factor (K) and threshold for oil impacts (β). Red and blue cells represent greatest and least potential impact, respectively. (PDF) [file pone.0190840.s015.pdf]

Sediment:WC concentration factor; rows: Oil effect threshold (ppb)

Snappers

Oil effect threshold (ppb)	1000	800	600	400
907	69%	71%	74%	76%
726	66%	69%	71%	74%
544	63%	66%	68%	72%
363	58%	61%	64%	66%

Large pelagic fish

Oil effect threshold (ppb)	1000	800	600	400
907	47%	51%	55%	61%
726	43%	47%	52%	58%
544	39%	42%	47%	53%
363	33%	36%	40%	46%

Groupers

Oil effect threshold (ppb)	1000	800	600	400
907	66%	68%	71%	74%
726	62%	66%	68%	72%
544	59%	62%	65%	69%
363	53%	56%	60%	64%

Small pelagic fish

Oil effect threshold (ppb)	1000	800	600	400
907	64%	66%	69%	71%
726	62%	64%	66%	69%
544	59%	61%	64%	67%
363	52%	55%	59%	63%

Drums and croakers

Oil effect threshold (ppb)	1000	800	600	400
907	63%	65%	68%	72%
726	60%	63%	66%	70%
544	56%	59%	63%	67%
363	51%	54%	57%	62%

Small demersal and reef fish

Oil effect threshold (ppb)	1000	800	600	400
907	37%	40%	44%	49%
726	34%	37%	41%	46%
544	29%	33%	37%	42%
363	24%	27%	31%	36%

Elasmobranchs

Oil effect threshold (ppb)	1000	800	600	400
907	69%	71%	74%	78%
726	66%	69%	72%	76%
544	62%	65%	68%	73%
363	56%	60%	63%	68%

Large demersal fish

Oil effect threshold (ppb)	1000	800	600	400
907	48%	51%	55%	61%
726	44%	48%	52%	58%
544	39%	43%	47%	53%
363	32%	36%	41%	47%

S2 Table. Sensitivity analysis showing the smallest observed biomass for various guilds relative to no-oil scenario. Biomass minima occur 7-16 months (median 10 months) after the oil spill. Parameters varied are sediment:water column concentration factor (K) and threshold for oil impacts (b). Red and blue cells represent greatest and least potential impact, respectively.
