# Supplementary material for: Impacts of the Deepwater Horizon oil spill evaluated using an end-to-end ecosystem model
Source: PLoS One. 2018 Jan 25;13(1):e0190840. doi: 10.1371/journal.pone.0190840 (PMC5784916; doi:10.1371/journal.pone.0190840)
Supplement: S3 Table — (PDF) [file pone.0190840.s016.pdf]

Taxa	Latin name	Reference
Red snapper**	*Lutjanus campechanus*	Allman, R., Barnett, B., Trowbridge, H., Goetz, L, and Evou, N. 2012. Red snapper (Lutjanus campechanus) otolith ageing summary for collection years 2009-2011. SEDAR31-DW05. SEDAR, North Charleston, SC. 26 pp
Vermillion snappers*	*Rhomboplites aurorubens*	Chih, C. 2015. Length and age frequency distributions for vermilion snappers collected in the Gulf of Mexico from 1981 to 2014. SEDAR 45-WP-08. November 2015. SEDAR, North Charleston, SC. 32 pp.
Gag	*Mycteroperca microlepis*	Lombardi, L., Fitzhugh, G. and Barnett, B. 2013. Age, length, and growth of gag (Mycteroperca microlepis) from the northeastern Gulf of Mexico: 1978-2012. SEDAR33-DW22. May 2013. SEDAR, North Charleston, SC. 26 pp.
Gray triggerfish*	*Balistes capriscus*	Lombardi, L., Allman, R., and Pacicco, A., 2015. Description of age data and estimated growth for Gray Triggerfish from the northern Gulf of Mexico: 2003-2013. SEDAR43-WP-010. March 2015. SEDAR, North Charleston, SC. 36 pp.
Red grouper	*Epinephelus morio*	Lombardi-Carlson, L. 2014. An age and growth description of Red Grouper (Epinephelus morio) from the northeastern Gulf of Mexico: 1978-2013 for SEDAR42. SEDAR 42-DW-10. SEDAR, North Charleston, SC. 39 pp.
Spanish mackerel**	*Scomberomorus maculatus*	Palmer, C., DeVries, D., Fioramonti, C. A review of Gulf of Mexico and Atlantis Spanish mackerel (Scomberomorus maculatus) age data, 1987-2011, from the Panama City Laboratory, Southeast Fisheries Science Center, NOAA Fisheries Service. SEDAR28-DW23. January 2012. SEDAR, North Charleston, SC. 26 pp.
King mackerel	*Scomberomorus cavalla*	Palmer, C., DeVries, D., Fioramonti, C. and Lang, H. 2013. A review of Gulf of Mexico and Atlantic king mackerel (Scomberomorus cavalla) age data, 1986 – 2013, from the Panama City Laboratory, Southeast Fisheries Science Center, NOAA Fisheries Service. SEDAR38-DW-15. SEDAR, North Charleston, SC. 59 pp.
Menhaden	*Brevoortia tyrannus*	SEDAR. 2013. SEDAR 32A - Gulf of Mexico menhaden Stock Assessment Report. SEDAR, North Charleston SC. 422 pp.

*VonBertalanffy model used to convert length to age

**Did not have 2012 data available; post spill based on 2011

S3 Table. References for SEDAR age structure data.
